# The educational effects of telemedicine training using role‐playing for general practice/family medicine residents: A qualitative study

**DOI:** 10.1002/jgf2.70020

**Published:** 2025-04-21

**Authors:** Koki Nakamura, Tomoo Hidaka, Yoshihiro Toyoda, Mei Endo, Satoshi Kanke

**Affiliations:** ^1^ Center for Medical Education and Career Development Fukushima Medical University Fukushima Japan; ^2^ Fukushima Centre for General Physicians Fukushima Medical University Fukushima Japan; ^3^ Department of Hygiene and Preventive Medicine Fukushima Medical University Fukushima Japan; ^4^ Department of General Internal Medicine and Family Medicine Fukushima Medical University Fukushima Japan

**Keywords:** family doctor, medical education, online medical consultation, open coding, role‐play

## Abstract

**Background:**

Despite the increasing demand for telemedicine, there have been few reports on telemedicine training for general practice/family medicine residents. This study aimed to qualitatively examine the educational effects of remote medical training for residents using role‐play.

**Methods:**

This study targeted first‐ and second‐year residents enrolled in the General Medicine/Family Medicine Residency Program at Fukushima Medical University in 2023. The residents watched educational videos on telemedicine and engaged in role‐playing training based on multiple scenarios. Subsequently, interviews were conducted with the residents, and the verbatim transcripts of the audio data were thematically analyzed using open coding.

**Results:**

Eight residents participated in the study, with each undergoing 3–4 interviews. The identified codes were inductively summarized, and nine categories were generated: verbal cues to enhance the quality of history taking, nonverbal communication to connect with patients, addressing risks inherent in the convenience of telemedicine, co‐creating a clinical environment with patients, anticipating issues different from those encountered in face‐to‐face consultation rooms, considering and supporting the elderly and those who are digitally disadvantaged, improving access restrictions due to busyness and resistance to telemedicine, understanding the living environment in connection with patient families and home care nurses, and awareness of the wide‐ranging applications of telemedicine.

**Conclusion:**

The results of telemedicine training via role‐play suggest various educational effects. This study provides crucial findings for considering educational methods for GM/FM residents to respond to the increasing demand for telemedicine in primary health care.

## 1 INTRODUCTION

Telemedicine refers to medical consultations conducted using telecommunication devices equipped with video call functions.^1^ Unlike audio‐only consultations, telemedicine allows for the incorporation of visual information that is critical to diagnosis, enabling physicians to assess patient expressions and make more precise clinical decisions. Additionally, visual contact with the physician's face may provide some patients with reassurance and enhance their understanding of explanations. Initially developed to provide care for patients in remote areas, telemedicine in primary health care proved its utility during the COVID‐19 pandemic by enabling care at a safe distance.^2^, ^3^ Today, it has become the “fourth mode of practice” alongside traditional outpatient, inpatient, and home care.^1^ The utility of telemedicine spans not only remote health care and treatment for acute infectious disease, but also the management of chronic diseases and mental health care for people who are busy with work and childcare.^4^ A systematic review investigating telemedicine in general practice/family medicine (GP/FM) reported that both physicians and patients found telemedicine to be convenient and practical.^5^ Therefore, the demand for telemedicine is expected to continue to increase in the future.

Adequate education for health care providers is essential to properly implement telemedicine. Medical schools are increasingly incorporating telemedicine training into their curricula, and the skills required to effectively provide telemedicine have also been proposed.^6^, ^7^, ^8^ However, despite lacking support to acquire these skills, GP/FM residents are expected to provide telemedicine to patients and instruct medical students in its practice.^9^ Therefore, providing appropriate telemedicine training opportunities to residents is critically important. The Japan Primary Care Association also recommends that GP/FM residents receive telemedicine training during their residency program.^10^ Despite this, a systematic review of telemedicine training for primary health care staff revealed very few reports on training for GP/FM residents.^11^ In addition, while some quantitative studies have shown that telemedicine training can positively impact residents' confidence, only one qualitative study to date has analyzed the specific insights and learning outcomes.^11^, ^12^

The purpose of this study was to qualitatively explore the insights and learning that residents gain after receiving telemedicine training.

## 2 METHODS

### 2.1 Participants

This study adopted purposive sampling and included eight first‐ and second‐year residents enrolled in the GP/FM Residency Program at Fukushima Medical University for the 2023 academic year. All of them were graduates of Fukushima Medical University, where they received lectures and clinical clerkships in GP/FM. All eight residents voluntarily participated in this study (hereinafter referred to as “participants”). In qualitative studies, it is challenging to establish clear criteria for sampling as in quantitative studies where sample sizes can be precisely calculated. However, empirical guidelines suggest that having 9 ± 2 participants is appropriate for categorizing experiences and obtaining transferable insights.^13^ Additionally, it was not feasible to involve residents from other programs on a continuous basis due to scheduling conflicts, preventing an increase in the number of participants. Under these conditions, conducting a thorough inductive analysis to derive meaningful insights for telemedicine training was the aim of the present study.

### 2.2 Training content

Telemedicine training was conducted from October 2023 to March 2024, once or twice a month, with one FM specialist/supervising physician using Zoom to train 2–3 participants per session. Initially, the participants watched a video called “Introduction to Online Diagnosis” created by the Japan Primary Care Association.^14^ This is learning content designed for primary care physicians to acquire communication skills and examination techniques unique to telemedicine through videos. Subsequently, using preprepared scenarios, the participants conducted telemedicine role‐plays among themselves (e.g., Participant A as the doctor, Participant B as the patient, and Participant C as the observer). The scenarios were based on everyday family medicine practice, consisting of 10 cases in total, with two cases each covering the common cold, lifestyle diseases, pediatric care, mental health, and geriatric care (Appendix S1). After the role‐play, the participant in the doctor role conducted a self‐assessment and received peer reviews from the participants in the patient and observer roles, as well as feedback from the supervising physician. This process was repeated for each session with different role assignments and scenarios, and each participant experienced the doctor role 3–4 times during the training period.

### 2.3 Data collection

Basic characteristics of the participants, such as age, gender, residency year, and experience with telemedicine, were collected. Immediately after each role‐play reflection, the supervising physician conducted interviews with the participant in the doctor role. Although the supervising physician and the participants were acquainted before this training, they did not have a relationship involving daily training evaluations. The interviews were conducted online in a semi‐structured format using an interview guide (Table 1), with each interview lasting approximately 15 min. With the participants' consent, the interviews were recorded, and verbatim transcripts of the audio data were created. It should be noted that the richness of data in qualitative study is obtained not by sample size but by ensuring multiple data collection opportunities, a viewpoint that has been validated in practice.^15^ In the present study, we interviewed eight people longitudinally, repeatedly, and in depth; This procedure allowed us to obtain a depth of data on the values, beliefs, and experiences held by the interviewees.

**TABLE 1:** Interview guide.

1. Did you gain any new perspectives or insights that you did not have before attending this training? For example, in terms of medical history taking, communication, or understanding the patient's context
2. Did you gain any new perspectives or insights from the peer review? For example, in terms of medical history taking, communication, or understanding the patient's context
3. Did you gain any new perspectives or insights from the feedback given by your supervising physician? For example, in terms of medical history taking, communication, or understanding the patient's context
4. Did you gain any new perspectives or insights unique to online consultations that differ from face‐to‐face consultations? For example, in terms of medical history taking, communication, or understanding the patient's context
5. How do you think you can apply the skills and insights gained from this training to actual online consultations with real patients, rather than role‐play scenarios? For example, in terms of medical history taking, communication, or understanding the patient's context

### 2.4 Data analysis

Thematic analysis was conducted using open coding based on the verbatim transcripts. Open coding is a qualitative and inductive coding method that involves assigning concise headings summarizing the content of the data.^16^ By progressively abstracting and categorizing the data, it is possible to organize the overall picture of the statements and more comprehensively analyze the experiences of the participants. This well‐established method is also used in medical fields.^17^ In the present study, the interview data were segmented into coherent units, and short phrases (codes) summarizing the content were assigned. The perspective of the educational effects of the training was set, and the content related to personal changes and growth before and after the training was extracted as units for analysis. To enhance the credibility of our qualitative analysis, we employed methodological triangulation. This method provides corroboration that the statements made in the interviews and the themes generated by the researcher's analysis do not deviate significantly from actual events. The telemedicine training in the present study was conducted via Zoom and was recorded on video. The participants' statements in the interviews were verified through observations of their responses to corresponding situations. As for member checking, researcher KN initially coded relevant sections from the interview transcripts, and researcher SK then reviewed and verified the coding to check for potential interpretive bias. The entire analysis process was supervised by researcher TH, an experienced qualitative researcher. All researchers collaborated to examine the procedures of stepwise abstraction, the appropriateness of labels and thematic nomenclature, and the possibility of alternative interpretations of the data. This was conducted across the entire scope of the analysis, and as a result, we determined that the study had reached saturation due to the depth of the data and analysis. Finally, the generated categories were overviewed, and a quadrant chart was created to organize the overall picture.

### 2.5 Ethics approval

This study was conducted with the approval of the General Ethics Committee of Fukushima Medical University (Approval Number: 2023‐044).

## 3 RESULTS

The participants in the study were 3 males and 5 females, aged either 27 or 28 years. The characteristics of the participants are shown in Table 2. None of the participants had any actual experience with telemedicine. Open coding generated 160 initial labels for the raw narratives, which were then consolidated into 38 codes and 21 subcategories, resulting in the final nine categories (Table 3). In addition, the parentheses at the end of the sentence indicate the participant's ID and which interview and question the narrative was taken from. For example, (E, 3, Q1) indicates that the narrative was taken from question 1 of the third interview with participant E.

**TABLE 2:** Participant characteristics.

ID	Age	Gender	Doctor years	Scenario in which they played the role of a doctor			
A	27	F	3	Common cold	Lifestyle diseases	Mental health	Geriatrics
B	28	M	4	Common cold	Pediatrics	Geriatrics	
C	28	M	4	Common cold	Lifestyle diseases	Mental health	Geriatrics
D	27	F	3	Common cold	Pediatrics	Mental health	
E	27	F	3	Lifestyle diseases	Pediatrics	Mental health	
F	28	F	4	Lifestyle diseases	Pediatrics	Geriatrics	
G	28	M	4	Lifestyle diseases	Pediatrics	Geriatrics	
H	28	F	4	Lifestyle diseases	Pediatrics	Geriatrics	

**TABLE 3:** Educational impacts of telemedicine training using role‐playing.

Categories	Subcategories	Codes	Narratives of participants
Co‐creating a clinical environment with patients	Preparation and confirmation of the setup for telemedicine	The need to proactively speak to patients when they are unaware of equipment problems, such as a screen not working	When it comes to issues like the screen not displaying properly, I think there's probably not much that can be done about mechanical problems. However, if the patient hasn't noticed the issue, it's important to proactively ask them questions like “Can you turn on your camera?” and actively communicate any points you've noticed to the patient. (E, 3, Q1)
		The need for doctors to prepare an environment suitable for telemedicine	This time, I might not have paid enough attention, but I think it was probably hard for you to see my face. The background was completely white, and my face appeared somewhat dark, making it difficult to see my expressions. I realized that I need to be mindful of how visible I am on the screen. Since this is not just a casual video call among friends, but rather something more formal, the impression of the camera is important. I need to ensure proper lighting in front of my face and consider how I appear on the camera. (G, 1, Q4)
	Understanding the overall process of telemedicine, including postconsultation procedures	Confirmation of the overall flow, including arrangements for telemedicine	I realized the importance of preparing for telemedicine, including the overall flow of the consultation and the necessary coordination with pharmacies, such as sending faxes. This training made me aware of the importance of such preparations. (G, 1, Q5)
		Awareness of the patient's unfamiliarity with the process of scheduling the next appointment and receiving medication	I realized that I hadn't explained the method for the next appointment or where to receive the medication. I'm concerned about whether the patients will understand, given that these methods are still not very familiar to them. If we can practice these aspects, I believe patients won't face any inconvenience during their consultations. (H, 1, Q5)
	Strategies to improve physical examination during telemedicine	Approaches for obtaining throat and respiratory findings online	I think one of the new things I learned today was about the unique ways to perform physical examinations in telemedicine. For example, shining a light to examine the throat or asking the patient to step back a bit to observe their breathing. (A, 1, Q1)
		Approaches for obtaining chest auscultation findings online: illustrating auscultation sites	I thought it might be useful to have a clear diagram showing where to place the stethoscope during auscultation. Having something like that to guide the patient, saying “Place it here,” would be helpful. It's hard to figure out the exact spot, especially over clothes. I found myself wanting a visual aid while giving instructions. (D, 1, Q1)
	Limitations of physical examinations via telemedicine	Difficulty in directing patients to adjust the lighting or brightness of the room to facilitate the examination	I didn't notice that the room was dim and the camera view was hard to see, so I continued without addressing it, thinking “What's going on here?” I should have asked the patient to turn on a light or brighten the room. It was challenging to get patients to move in ways I naturally would during an in‐person consultation. This aspect is unique to telemedicine, where patients need to actively participate more, which was quite difficult. (E, 2, Q4)
		Difficulty in obtaining physical findings in children, which requires more effort compared with adults	I believe it can be challenging to conduct examinations through the screen for adults, as there are more indirect steps involved. However, with children, the process can be even more complicated, like a game of telephone through their parents, making it difficult to quickly obtain physical findings. For instance, getting a child to show their throat may involve first getting them to come close to the screen, and some might not even show their face at all. (H, 2, Q1)

	Challenges of telemedicine for children	Children have unique challenges, such as difficulty staying still in front of the camera and mischievous behavior	Considering actual telemedicine consultations with children, it's clear that getting them to sit properly in front of the camera is unlikely. Even if you manage to get them in front of the camera, they might reach for a phone or play with something, making it hard to conduct a proper examination. It seems more challenging to listen to their concerns and perform the consultation effectively compared with a regular consultation room setting. (F, 2, Q1)
Concerns about the legal implications, such as child pornography laws, when imaging a child's genital area, even for medical purposes		I was a bit concerned that asking to show a child's genital area might legally conflict with certain regulations. Even if it's for medical purposes, asking to show the whole body, including the genital area and possibly the breasts, could raise questions like, “Is this allowed?” I think it would be wise to establish clear guidelines within the clinic about what can be shown. (G, 2, Q3)
Verbal cues to enhance the quality of history taking	Universality of medical history taking and approaches to complement physical examinations in telemedicine	The realization that medical history taking in telemedicine is not significantly different from face‐to‐face consultations	Regarding medical history, I don't think it changes much in an online setting. There's no need to alter the approach just because it's telemedicine, so I feel that conducting it as usual should be fine. (B, 2, Q4)
		The importance of interviews to compensate for unclear images	I realized that I should have asked more detailed questions about the images and the characteristics of the rash. I should have asked things like “What kind of rash is it?” and “Are there any blisters?” each time. (D, 2, Q4)
	Difficulty in grasping a patient's first impression and overall condition during telemedicine	The inability to observe the patient's entry into the examination room in telemedicine, unlike in‐person consultations	In face‐to‐face consultations, I usually observe whether the patient walks in by themselves or uses a wheelchair. However, in telemedicine, this is something I cannot see, so I miss that initial impression. It's difficult to judge how the patient appears at first glance and whether they look unwell or healthy just from the screen. (E, 1, Q1)
		Verbal collection of information online regarding movements, which can be obtained instantly in face‐to‐face consultations	The biggest issue, I think, is that I can't see how the patient is living. In a home visit, I can quickly gather information about how they spend their time in their room or if they can come to greet me. But with telemedicine, I can only see what's in front of the screen. It's difficult to pick up on everyday details from words alone. You need to spend time asking questions to gather such information, which makes it challenging in an online setting. (G, 3, Q4)
Nonverbal communication to connect with patients	Points to note when communicating online	The need for careful consideration in communication with patients online due to the difference in emotional distance compared with face‐to‐face consultations	When I asked patients about their family circumstances, I realized that my offhand comments might not align with the patient's feelings or intentions. This made me aware that in telemedicine, there is an unseen emotional distance, and I should be cautious about making such remarks. This was a significant realization for me. (C, 1, Q3)
		The realization that verbal acknowledgments may disrupt the flow of telemedicine consultations	Regarding giving verbal cues like “uh‐huh” or “I see,” which I would say casually during face‐to‐face consultations, I realized that in telemedicine, they might be interpreted as interruptions. I think I need to refine such communication aspects unique to telemedicine. (C, 2, Q4)
		The possibility that a lack of eye contact can hinder the establishment of trust and lead to a decrease in clinical performance	When a patient keeps looking down, it makes me think they might not be opening up, which makes the situation a bit challenging. It would be better if our eyes met frequently, but when they don't, I can't understand the reason due to the limited camera angle. In person, I would be able to see if the patient is just looking down or fiddling with their phone, but online, I can't see that, so I end up speculating and might feel negative emotions, which could reduce my performance. (C, 3, Q4)
	
	Difficulty in balancing the explanation of materials and checking the patient's understanding during telemedicine	When explaining something, my attention tends to be entirely on the materials, noting “This is written here” and focusing on the documents. It might be challenging to gauge the patient's understanding of those materials through nonverbal cues. (C, 3, Q4)
	Improving communication in telemedicine	Differences in spacing between online and face‐to‐face consultations, and allowing more pauses in online consultations	I found it challenging to manage the slight time lag and the timing of pauses. It's about avoiding talking over the other person. I think it's necessary to consciously leave slightly longer pauses than usual and be more aware of these aspects. (B, 1, Q4)
Strategies to make it clear who is being spoken to in online consultations with multiple participants		When there are multiple participants, such as the patient's family, it can be unclear who I'm speaking to online. This was pointed out to me, and I realized that I need to be more mindful of it in the future. I think it would be better to address the person by name before speaking to them. (B, 3, Q5)
Addressing risks inherent in the convenience of telemedicine	The convenience of telemedicine and understanding of patient's context	The patient's context for choosing telemedicine	In a regular consultation room, I often ask patients, “Is there a particular reason you decided to come in for a consultation today?” When I ask, “Why did you choose to have an online consultation today?” it might reveal that the patient has reasons for not being able to come in person. This could include being busy with childcare or work, and it might provide insights into their background. (A, 1, Q4)
		Insufficient understanding of patient's context due to the convenience patients desire	When a patient comes in wanting to consult and discuss, I can listen to them thoroughly. However, some patients might be too busy and prefer to keep the telemedicine session as short as possible just to get their prescription. In such cases, I worry that we might not address the underlying issues they are facing. When patients take the time to come to the hospital, they are somewhat committed to discussing their issues. But for those aiming to make telemedicine appointments as short as possible, it might be challenging to delve into the full context of their problems. (D, 1, Q3)
		How to convey the ease of online medical care and when face‐to‐face consultation is necessary	Many of the people who use telemedicine are those who just want medication or relief from their symptoms. I realized that I need to mention when it's truly necessary for them to visit the hospital in person, which is a bit different from my usual approach in regular consultations. (D, 1, Q1)
		The reduced barriers to suggesting follow‐up consultations in telemedicine compared with face‐to‐face consultations	I realized that I need to be mindful about setting up a safety net. Specifically, I should be more stringent in scheduling follow‐up appointments. Since we can't monitor their condition as easily as we do in face‐to‐face consultations, it's important to ask them to come in earlier if they show any symptoms. (B, 1, Q1)
		Inadequate context understanding in telemedicine leading to cautious patient communication	I realized that not fully understanding the context could lead to an inadvertent remark meant to be kind, which might actually hurt the patient. This makes me hesitant to speak up. (C, 1, Q3)
	Benefits of telemedicine for children	Reduced infection risk with telemedicine for pediatric patients with regular prescriptions	If it's just a routine visit to get regular medication and nothing has changed, then the mother won't have to take her child to a clinic full of kids with colds. It saves time and seems like a good idea. (F, 2, Q1)
Improving access restrictions due to busyness and resistance to telemedicine	Benefits of telemedicine for patients with lifestyle‐related diseases	Improving accessibility for busy patients with lifestyle‐related diseases	For busy working individuals, I've had regular patients who sometimes miss appointments unexpectedly, leaving me wondering why they didn't show up. I think creating an environment where they can easily attend regular check‐ups is one of the benefits of telemedicine. Instead of thinking, “I can't make it today, so never mind,” they might feel, “I can spare 10 min for an online consultation.” This lowers the barrier to continuing their visits. (A, 2, Q4)
	The benefits of telemedicine mental health consultations	Improving accessibility for mental health patients	In cases involving mental health or when privacy is a concern, being able to connect from home through telemedicine might lower the barrier for those who feel reluctant to visit a hospital for their symptoms. Especially for those with time constraints, telemedicine could make it easier for them to seek help. (A, 2, Q4)
Understanding the living environment in connection with patient families and home care nurses	Utilizing telemedicine with family participation	The usefulness of family participation in telemedicine	I think having family members participate in telemedicine sessions is crucial. Many conditions require family support, so knowing that telemedicine can involve the family is a highly effective approach. This is especially true for those who want to continue using telemedicine in the future. (E, 3, Q1)
	Utilizing telemedicine for home medical care	Collaboration between telemedicine and visiting nursing	I wonder if it could be used in home nursing visits. For instance, when a nurse visits and finds something potentially concerning, they might connect with a doctor to examine the situation together. I thought that if it were used for elderly patients, having a nurse present could help ensure better auscultation than a regular person might achieve. I wondered if such a use case might emerge. (D, 1, Q1)
		The mental state of a mother, inferred from the tidiness of the house observed through the online consultation	It's not limited to pediatrics, but being able to see the home environment provides insights. For example, seeing if the house is well‐kept can reflect the family's overall state of affairs. There are people who can keep their homes tidy and those who can't. The state of the home might give clues about the mother's mental state. If I saw a cluttered room in the background and the parent looked exhausted, I might have said, “It must be tough for you.” (F, 2, Q4)
Anticipating issues different from those encountered in face‐to‐face consultation rooms	Issues with ensuring privacy in telemedicine	Easier privacy protection in face‐to‐face consultations depending on the situation	On the other hand, there might be times when patients can't talk openly because there are people at home, or if they're connecting from work, they might not want their colleagues to overhear. In a one‐on‐one consultation room setting, patients might feel more comfortable talking, but they might not want to discuss things when surrounded by others at home. (D, 1, Q3)
	Suspension of telemedicine	Difficulty in dealing with patients who leave their seats mid‐way	There was a moment when the patient left, and I wasn't sure what to do. I told them to let me know when they returned, but I wasn't sure if I should proactively ask if they were back or just wait. Timing these interactions is one of the challenges unique to telemedicine, I think. (E, 3, Q4)
	Challenges of online mental health consultations	Difficulty in assessing the patient's depression and mood in mental health telemedicine	For mental health patients using telemedicine, feelings of depression can make it challenging. The lack of direct eye contact makes it harder to convey the atmosphere of the session. There's a sense of disconnect compared with in‐person interactions, where the exchange of energy and the use of silent moments play a significant role. It feels difficult to capture that in an online setting. (C, 3, Q1)
Considering and supporting the elderly and those who are digitally disadvantaged	Consideration and support for the elderly and the digitally disadvantaged	The unfamiliarity of elderly patients with online devices and the need for personnel to assist with operation	From the perspective of telemedicine for elderly patients, I realized during the process that they are not familiar with basic things like operating the equipment or balancing the volume. This scenario involved a visiting nurse who likely took care of such settings to some extent. However, if there were no support staff to help when visiting a patient's home, explaining and setting up the equipment from scratch might be quite challenging. (C, 4, Q1)
		The decline in hearing and vision in the elderly that is crucial for online medical consultations	For elderly patients, some have hearing difficulties. When visiting them at a facility, conversations often involve getting very close to their ear. I wonder if this can be managed online. There are also patients with cataracts who can't see well. Telemedicine relies on both sight and hearing being functional. (A, 4, Q5)
Awareness of the wide‐ranging applications of telemedicine	Applying online training to face‐to‐face consultations	Improvement in face‐to‐face consultations through overcoming challenges in telemedicine	(As you become better at understanding context in telemedicine, it can also improve your skills in face‐to‐face consultations. Can telemedicine training enhance in‐person consultations?) Yes, I think so. It will likely sharpen those skills even more. (C, 1, Q5)
	Utilizing telemedicine for younger generations	Differences in age groups of patients between face‐to‐face and online consultations	I think the age demographics might vary. It seems like those who use smartphones, mainly in their 50s and 60s, might be the ones who would opt for telemedicine. These would be individuals who are too busy to visit in person, such as those occupied with work or household chores. This could mean that the patient demographics for telemedicine are somewhat different from regular consultations. (D, 1, Q4)

*Note*: The parentheses at the end of the narratives of participants indicate the participant's ID and which interview and question the narrative was taken from. For example, (E, 3, Q1) indicates that the narrative was taken from question 1 of the third interview with participant E.

### 3.1 Verbal cues to enhance the quality of history taking

The participants emphasized the universality of history taking and its importance in supplementing physical examinations, even in telemedicine. They noted that it is more challenging to assess the patient's initial physical appearance and overall condition in telemedicine compared with face‐to‐face consultations. Specifically, in outpatient consultations, the participants intuitively grasped each patient's condition by observing the patients as they entered the consultation room. Similarly, in home visits, they formed an initial impression based on the patient's living environment. On the contrary, in telemedicine, since patients start the consultation already positioned in front of a computer screen, the study participants realized that they could not quickly grasp contextual information about the patient. In addition, they learned that such information needs to be supplemented through history taking and that utilizing verbal cues is crucial for eliciting it. (G, 3, Q4).

### 3.2 Nonverbal communication to connect with patients

The participants discussed the unique communication considerations specific to telemedicine, which differ from face‐to‐face consultations. They emphasized the need to be more cautious with verbal communication due to the perceived difference in emotional distance between online and in‐person interactions. (C, 1, Q3) They also gained some ideas for improving online communication. (B, 3, Q5).

### 3.3 Addressing risks inherent in the convenience of telemedicine

The participants realized that because patients perceive online consultations as convenient, there are differences in communicating the need for face‐to‐face consultations and follow‐up appointment instructions compared with in‐person visits. (D, 1, Q1) The participants also recognized that their preconceived notion that patients want convenience in online consultations tends to lead to a lack of understanding of the context. (D, 1, Q3).

### 3.4 Co‐creating a clinical environment with patients

The participants gained an understanding of the overall process of online consultations, including preconsultation preparations and postconsultation arrangements. (E, 3, Q1) They also discussed the challenges and limitations of online physical examinations, with particular concerns raised in pediatric care. (E, 2, Q4) (F, 2, Q1).

### 3.5 Anticipating issues different from those encountered in face‐to‐face consultation rooms

The participants recognized that online consultations could present distinct issues from those in the face‐to‐face consultation room, especially the possibility of interruptions. (E, 3, Q4).

### 3.6 Considering and supporting the elderly and those who are digitally disadvantaged

The participants spoke of the need for consideration and support for the elderly and those unfamiliar with digital devices. In particular, they noticed the contradiction that vision and hearing, which are key to online medical care, tend to decline among the elderly. (A, 4, Q5).

### 3.7 Improving access restrictions due to busyness and resistance to telemedicine

The participants noted that online consultations improve accessibility for patients, especially those who require care for lifestyle‐related diseases, mental health issues, and pediatric illnesses. (A, 2, Q4).

### 3.8 Understanding the living environment in connection with patient families and home care nurses

The participants mentioned the benefits of involving family members and visiting nurses in online medical consultations. In particular, they mentioned specific situations regarding collaboration with visiting nurses. (D, 1, Q5).

### 3.9 Awareness of the wide‐ranging applications of telemedicine

The participants saw the potential for online consultations to be useful for the younger generation and were aware that overcoming the challenges of online consultations would also contribute to improving their face‐to‐face consultation skills. (C, 1, Q5).

**FIGURE 1:**
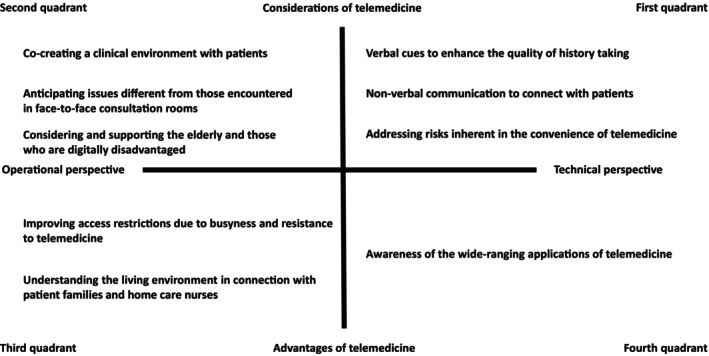
A quadrant diagram of the realizations and learnings gained by participants through telemedicine training. The horizontal axis represents the technical/operational perspective, and the vertical axis represents the advantages/considerations of telemedicine. To clarify, the first quadrant represents the technical perspective and considerations, the second quadrant represents the operational perspective and considerations, the third quadrant represents the operational perspective and advantages, and the fourth quadrant represents the technical perspective and advantages.

Upon reviewing and generating the categories, it was found that the overall picture could be organized along two dimensions: technical/operational perspectives and the advantages/considerations of telemedicine, leading to the creation of a quadrant chart (Figure 1). The first quadrant (technical perspective, considerations) included three categories: enhancing the quality of medical interviews through verbal communication, nonverbal communication to connect with patients, and addressing the risks inherent in the convenience of telemedicine. The second quadrant (operational perspective, considerations) also included three categories: co‐creating a clinical environment with patients, anticipating issues different from those in consultation rooms, and considering and supporting both the elderly and those with digital divides. The third quadrant (operational perspective, advantages) included improving access restrictions due to busyness and resistance to telemedicine, and understanding the living environment connected with patient families and home care. The fourth quadrant (technical perspective, advantages) included awareness of the wide‐ranging applications of telemedicine.

## 4 DISCUSSION

In the present study, we analyzed verbatim transcripts of interviews with participants to qualitatively explore the insights and learning they gained through telemedicine training. The qualitative analysis yielded nine categories reflecting the participants' insights and learning. By organizing these categories into a quadrant chart based on two dimensions, technical/operational perspectives and the advantages/considerations of telemedicine, we obtained an overall picture of the training's effectiveness. Below, we discuss the findings based on the quadrant chart.

In the first quadrant (technical perspective, considerations), the participants mentioned the universality of medical interviews, even in telemedicine. This is consistent with the results of previous research, which indicate that most residents were able to obtain basic history, such as current illness as well as surgical and social history, during telemedicine consultations.^12^ Additionally, the participants in the present study highlighted the importance of medical interviews in supplementing physical examinations. Notably, they pointed out the necessity of using verbal communication to gather information about the patient's initial physical appearance and overall condition, which are more challenging to grasp in telemedicine compared with face‐to‐face consultations.

The participants also discussed unique communication considerations specific to telemedicine, different from those in face‐to‐face consultations. Prior research has suggested that the online environment itself poses challenges to the traditional communication model between patients and physicians.^18^ A new insight was that the perceived emotional distance in telemedicine made participants more cautious in their verbal communication with patients. The participants recognized that assuming patients find telemedicine convenient might hinder the suggestion of face‐to‐face consultations or result in a lack of understanding of the patient's context. Telemedicine typically provides better access to the patient's context since they can attend consultations from home. However, interestingly, previous research found that residents were less likely to obtain information about the patient's home environment during telemedicine consultations compared with face‐to‐face settings.^12^ The results of the present study suggest that recognizing such preconceptions and leveraging the online environment to understand the context are crucial when conducting telemedicine.

In the second quadrant (operational perspective, considerations), the participants gained insights into the overall flow of preparing for telemedicine sessions and managing postconsultation tasks. Previous research has also shown that few residents were able to effectively explain telemedicine platforms to patients or evaluate whether their patients understood how to use them and what concerns they had about virtual care.^12^ Although the participants in the present study largely belong to the digital native generation, this background does not in itself guarantee competence in the implementation of telemedicine or in online communication with patients.^19^ Therefore, it is beneficial to provide the opportunity to review technical aspects during training before engaging in telemedicine with patients. The participants learned that physical examinations in telemedicine require a greater degree of patient cooperation. Previous research showed that no residents conducted physical examinations in the online environment or discussed methods of physical examinations with patients.^12^ In the present study, the participants were able to apply the scenes of physical examinations from the educational videos they watched just before the role‐play.^15^ However, the participants also expressed concerns about the limitations of physical examinations in telemedicine, particularly in pediatric care. This finding is novel and was gained through role‐plays involving multiple scenarios, including those with children. Next, the participants acknowledged that telemedicine could present different issues compared to those encountered in regular examination rooms. By including a scene where the patient leaves to take care of their pet in certain scenarios, the participants experienced interruptions, which are unlikely in a typical face‐to‐face setting. Given that interruptions such as having visitors or attending to children can happen during actual telemedicine, this was considered a valuable insight for the participants. Furthermore, the participants highlighted the need to consider and support elderly patients and those who are digitally disadvantaged. They particularly noted the contradiction that telemedicine relies on visual and auditory abilities, which tend to be diminished in elderly patients.

In the third quadrant (operational perspective, advantages), the participants noted that telemedicine particularly improves accessibility for patients requiring care in lifestyle‐related diseases, mental health, and pediatric medicine. This finding is in line with a systematic review that reported patients find telemedicine convenient in GM/FM.^5^ Additionally, the participants highlighted the usefulness of involving families and home care nurses in telemedicine. They specifically mentioned concrete examples of collaboration with home care nurses. In Japan, telemedicine (Doctor to Patient with Nurse [D to P with N]) involving nurses and patients in rural areas has already been initiated.^20^

In the fourth quadrant (technical perspective, advantages), the participants discovered the potential for telemedicine to be effectively utilized by younger generations. Additionally, they recognized that overcoming the challenges of telemedicine could also enhance their proficiency in face‐to‐face consultations. Previous research has identified unique challenges specific to online training by conducting and comparing identical scenarios in both face‐to‐face and online settings.^12^ However, the realization in the present study of applying the insights gained from telemedicine training to improve face‐to‐face consultations is a novel perspective.

This study has several limitations. Firstly, the patient role in the role‐play was performed by fellow residents rather than standardized patients. This lack of standardization may have influenced the learning and insights of the residents in the doctor role. Secondly, the training consisted of role‐playing exercises, which are only a part of actual telemedicine practice. To conduct actual telemedicine, further training is needed in areas such as billing procedures and how to issue prescriptions. Additionally, it is noteworthy that the setting was a controlled environment, and thus, real patients and their real‐life environments were not used in the present study.

## 5 CONCLUSION

Through the telemedicine training using role‐play, residents gained various insights and learnings. This study provides crucial findings for considering educational methods for GM/FM residents to respond to the increasing demand for telemedicine in primary health care.

## AUTHOR CONTRIBUTIONS

**Koki Nakamura:** Conceptualization; investigation; writing – original draft; methodology; formal analysis; project administration; data curation; visualization; resources. **Tomoo Hidaka:** Writing – review and editing; conceptualization; methodology; supervision; visualization. **Yoshihiro Toyoda:** Investigation; resources. **Mei Endo:** Investigation; resources. **Satoshi Kanke:** Formal analysis; supervision.

## CONFLICT OF INTEREST STATEMENT

The authors have stated explicitly that there are no conflicts of interest in connection with this article.

## ETHICS STATEMENT

Ethics approval statement: This study was conducted with the approval of the General Ethics Committee of Fukushima Medical University (Approval Number: 2023‐044).

Patient consent statement: None.

Clinical trial registration: None.

## PERMISSION TO REPRODUCE MATERIAL FROM OTHER SOURCES

None.

## Supporting information


Appendix S1

## Data Availability

The datasets used and/or analyzed during the current study are available from the corresponding author upon request.
