# Supplementary material for: The educational effects of telemedicine training using role‐playing for general practice/family medicine residents: A qualitative study
Source: J Gen Fam Med. 2025 Apr 21;26(4):334–45. doi: 10.1002/jgf2.70020 (PMC12237810; doi:10.1002/jgf2.70020)
Supplement: Supplementary file 1 — Appendix S1 [file JGF2-26-334-s001.docx]

**全て架空の事例です(All are fictitious cases)**

2023/10

感冒シナリオ①：**医師役**シート

**課題**

あなたはH町にある無床診療所の医師です。コロナ禍の収束後もオンライン診療に取り組んでいます。オンラインの診察でも専用機器を用いて胸部の聴診は可能です。

平日の午前中、次の患者をオンラインで診療することになりました。

**患者：28歳　女性　鹿島くるみ さん**

**主訴：咳、鼻汁**

バイタルサイン：血圧120/70 mmHg、脈拍58/分（義父母の家庭血圧計で測定）、

体温 36.2℃、SpO2 99％（コロナ禍に購入したパルスオキシメーターで測定）

既往歴：特記なし

アレルギー歴：なし

喫煙歴：なし

飲酒歴：なし

この患者の診療を⾏ってください。時間は**10分間**です。

シナリオ①：**患者役**シート

**課題**

あなたはH町にある無床診療所の医師です。コロナ禍の収束後もオンライン診療に取り組んでいます。オンラインの診察でも専用機器を用いて胸部の聴診は可能です。

平日の午前中、次の患者をオンラインで診療することになりました。

**患者：28歳　女性　鹿島くるみ さん**

**主訴：咳、鼻汁**

バイタルサイン：血圧120/70 mmHg、脈拍58/分（義父母の家庭血圧計で測定）、

体温 36.2℃、SpO2 99％（コロナ禍に購入したパルスオキシメーターで測定）

既往歴：特記なし

アレルギー歴：なし

喫煙歴：なし

飲酒歴：なし

この患者の診療を⾏ってください。時間は**10分間**です。

**〜雰囲気〜 　表情、声には力がない**

・医師役からの「今日はどうされましたか？」という質問に対して

**今朝起きた時から鼻水と咳が少し出てて…。子供たちの風邪がうつったんだと思います。**

**だるさもあって、なんだか疲れてしまいました…。**

・以下は医師役からの質問や会話の流れに合わせて

4日前から上の子（長男3歳）が熱と咳を発症、近くの小児科を受診、風邪の診断で薬で治療中。

3日前から下の子（次男8ヶ月）も熱を出した。二人とも徐々に治ってきていて体調は心配ないが、不機嫌になったり、夜に咳き込むため、育児・看病で疲れがたまってきていた。

昨日の日中は寝不足でだるかった、夕方に喉がイガイガしてきた。熱はなかった。

今日の起床時から鼻水と咳が少し出ていて、だるさが増している。食欲もあまりない。体温は自宅で測ると36.7℃、平熱が35℃台なので普段より高めだった。夜勤明けの夫に子どもをみてもらっている間にオンラインで診てもらおうと思って受診した。

悪寒戦慄なし、唾液の流涎なし、呼吸困難なし、喘鳴なし。

周囲の流行歴：地域にCOVID-19、インフルエンザの流行はない。長男の保育園では風邪で休む子どもがちらほら出ている。人が集まる場所への外出や最近の海外渡航歴はない。

妊娠の可能性：なし（ 授乳中）

職業：育休中（産休前は事務職）

家族：夫、子供2人（長男3歳、次男0歳8ヶ月）、義理の両親と6人暮らし。夫は鉄道会社勤務で、駅員をしており夜勤もある。義理の両親は高血圧で当院通院中、子供たちが風邪をひいている時は感染対策のため生活を分けている。実の両親は他県（車で3時間）にいる。

・身体所見

身長148cm、体重42kg、胸部：心音整、心雑音なし、ラ音なし。

・かきかえ

解釈：子供たちから移った風邪だろう。だるいのは育児疲れや寝不足も重なっている。

期待：だるさをとってほしい。本当に期待していることは「ただただ休みたい」。

感情：辛い、疲れた。夫は今晩も夜勤のため、夜ひとりで子どもたちの面倒をみれるか不安です。義父母は感染対策のため子供が風邪引いていると育児を頼めない（→義父母にはこういうときこそ子育てを手伝ってほしいのに…）。

影響：だるくて横になっていたい。家事・育児も義務感でやろうと思うが、辛い。

【解説】

・子育て世代は、自分のみならず子供の健康管理も必要。ひとたび子供が風邪を引くと家族全体の健康問題へと発展する。夫婦や親世代の育児への関わり方にもよるが、一般的に母親に育児・看病の負担が集中し、疲労やストレスが高まりやすい。

・このような家族のシステムに目を向け、健康問題を把握すると共に、家族のリソースを考慮したケアの方針相談ができるかが『家庭医の腕の見せどころ』である。

・地域によっては、病児・病後児預かりといった社会的サポートにつなげることもできる。

<https://www.city.fukushima.fukushima.jp/kseisaku-shien/kosodate/kosodate/shien/seido08.html>

・うつ病も気になるので、フォローアップやセーフティーネットを張ることも重要である。

2023/10

感冒シナリオ②：**医師役**シート

**課題**

あなたはH町にある無床診療所の医師です。コロナ禍の収束後もオンライン診療に取り組んでいます。オンラインの診察でも専用機器を用いて胸部の聴診は可能です。

平日の午前中、次の患者をオンラインで診療することになりました。

**患者：37歳　男性　花見 潤 さん**

**主訴：咳、鼻汁**

バイタルサイン：血圧148/88 mmHg、脈拍58/分（家庭血圧計で測定）、

体温 36.2℃、SpO2 99％（コロナ禍に購入したパルスオキシメーターで測定）

既往歴：高血圧で当院通院中、入院・手術歴なし。

アレルギー歴：特記なし

喫煙歴：なし

飲酒歴：なし

この患者の診療を⾏ってください。時間は**10分間**です。

感冒シナリオ②：**患者役**シート

**課題**

あなたはH町にある無床診療所の医師です。コロナ禍の収束後もオンライン診療に取り組んでいます。オンラインの診察でも専用機器を用いて胸部の聴診は可能です。

平日の午前中、次の患者をオンラインで診療することになりました。

**患者：37歳　男性　花見 潤 さん**

**主訴：咳、鼻汁**

バイタルサイン：血圧148/88 mmHg、脈拍58/分、体温 36.2℃、SpO2 99％（職場の保健室で測定）

既往歴：高血圧で当院通院中、入院・手術歴なし。

アレルギー歴：特記なし

喫煙歴：なし

飲酒歴：なし

この患者の診療を⾏ってください。時間は**10分間**です。

**〜雰囲気〜 　だるそうな様子はない**

・医師役からの「今日はどうされましたか？」という質問に対して

**今朝起きた時から鼻水が少し出ていたのですが、だるさもなく仕事に行きました。出勤してからはチョロチョロ鼻が出てきて頻繁に鼻をかむようになりました。鼻水が喉に降りてくると気持ち悪く、咳も少しでてました。**

・以下は医師役からの質問や会話の流れに合わせて

1日前：体調いつも通り。

当日：起床時から透明でサラサラした鼻汁が少し出ててるが、倦怠感なし。いつも通り出勤した。勤務中に絶えず鼻水が出てきて、頻繁に鼻をかむため仕事に集中しづらい。次第に喉にもたれてくるようになり喉の奥が不快になってきている。咳払いも少々あり。その他体調は悪くないが、鼻水をどうにかしたいと思った。仕事の合間に、職場の保健室からオンラインで診てもらおうと思った。

悪寒戦慄なし、唾液の流涎なし、呼吸困難なし、喘鳴なし。

周囲の流行歴：地域にCOVID-19、インフルエンザの流行はない。職場に風邪の人はいない。人が集まる場所への外出や最近の海外渡航歴はない。

内服薬：アムロジピン 5mg 1T 1×朝食後

健診歴：職場健診を毎年受けている。血圧と肥満の他、明らかな異常なし。

職業：事務職（弁護士事務所）

家族：独身、一人暮らし。両親は他県にいる。

・身体所見

身長165cm、体重75kg、顔：前額部、頬部に疼痛なし、胸部：心音整、心雑音なし、ラ音なし。普段の家庭血圧：130-140/80-90mmHg

・かきかえ

解釈：風邪だろうけど、また副鼻腔炎になるかもしれない。もうなってるのかも？風邪に抗菌薬が効かないことは知っているが、副鼻腔炎なら抗菌薬を処方してもらったほうがいいのかな。

期待：鼻水を止めてほしい。これは副鼻腔炎なのか知りたい。

感情：鼻水は煩わしい。前みたいに副鼻腔炎で長引くのは嫌だ。（より具体的な心配を聞かれたり、「他に心配なことは？」と深掘りされたら）前に副鼻腔炎になったときは鼻が詰まって夜も眠れなくてかなり辛い思いをした。

影響：鼻水のせいで、仕事に集中力を欠く。夜も寝れないかもしれない。（より具体的に聞かれたら）長い文章を読んだり書いたりするので、鼻をかんだり・すすったりする度に気が散って困る。

【解説】

・鼻汁、鼻閉症状は医学的には軽微な臨床症状だが、QOLを損なうため患者自身にとって重要度の高い症状となりうる。

・具体的には業務やスポーツのパフォーマンスが落ちたり、睡眠の質を低下させたりする。

・症状の感じ方は人それぞれであり、個々人の病気の経験を具体的に受け止めながら、対症療法や療養指導をオーダーメイドしていくところが『家庭医の腕の見せどころ』である。

・「このようになったら来院（対面診療）するように」とセーフティーネットを張ることも重要である。文

2023/11

生活習慣病シナリオ①：**医師役**シート

**課題**

あなたはH町にある無床診療所の医師です。コロナ禍の収束後もオンライン診療に取り組んでいます。オンラインの診察でも専用機器を用いて胸部の聴診は可能です。

平日の午後（15時頃）、次の患者をオンラインで診療することになりました。

**患者：56歳　女性　高井敦子 さん**

**受診理由：高血圧の定期受診**

X年7 月の職場健診で血圧高値（1 回目：164/102mmHg、 2 回目：154/94mmHg）の指摘あり、同年10 月 17 日に当院を初診した。生活習慣改善の指導と家庭血圧測定を指示した。11 月 14 日再診、生活習慣改善に真面目に取り組めたが、平均家庭血圧：142/80mmHg(顕著な日内変動なし)、診察時血圧：148/82mmHg と降圧不十分であった。もう少しがんばりたいとの本人の意向であったため、１ヶ月後再診とした。12 月 19 日再診、引き続き生活習慣改善に取り組んでいたが、平均家庭血圧：144/82mmHg、診察時血圧：146/85mmHg と降圧不十分であった。エナラプリル(5 ㎎)1 錠 1 回朝食後を開始した。

X+1年1 月 17 日再診、内服の飲み忘れは皆無で、平均家庭血圧：124/72mmHg、診察時血圧：126/74mmHg と改善を認めたため、同処方を継続とし、次回の定期受診は出版会社が忙しい時期だったので職場からオンラインで診療することとした。本日（2月20日）、予定通りオンライン診療をこれから実施する。尚、これまでの診察は毎回、今回の医師役が対応している（今回で4回目）。

【既往歴】特記すべきことなし

【アレルギー歴】特記なし

【生活歴】飲酒：なし、喫煙：なし、仕事：出版会社で雑誌の編集をしている。

【家族歴】父：高血圧(当院通院中)、母：脂質異常症(当院通院中) 、両親と3人暮らし。

【身体所見】(初診時) 身長：160 ㎝、体重：54 ㎏、胸腹部診察：異常なし

【検査】(初診時) 血液：異常なし、心電図：異常なし、胸部 X 線：異常なし

この患者の診療を⾏ってください。時間は**10分間**です。

2023/11

シナリオ①：**患者役**シート

**課題**

あなたはH町にある無床診療所の医師です。コロナ禍の収束後もオンライン診療に取り組んでいます。オンラインの診察でも専用機器を用いて胸部の聴診は可能です。

平日の午後（15時頃）、次の患者をオンラインで診療することになりました。

**患者：56歳　女性　高井敦子 さん**

**受診理由：高血圧の定期受診**

X年7 月の職場健診で血圧高値（1 回目：164/102mmHg、 2 回目：154/94mmHg）の指摘あり、同年10 月 17 日に当院を初診した。生活習慣改善の指導と家庭血圧測定を指示した。11 月 14 日再診、生活習慣改善に真面目に取り組めたが、平均家庭血圧：142/80mmHg(顕著な日内変動なし)、診察時血圧：148/82mmHg と降圧不十分であった。もう少しがんばりたいとの本人の意向であったため、１ヶ月後再診とした。12 月 19 日再診、引き続き生活習慣改善に取り組んでいたが、平均家庭血圧：144/82mmHg、診察時血圧：146/85mmHg と降圧不十分であった。エナラプリル(5 ㎎)1 錠 1 回朝食後を開始した。

X+1年1 月 17 日再診、内服の飲み忘れは皆無で、平均家庭血圧：124/72mmHg、診察時血圧：126/74mmHg と改善を認めたため、同処方を継続とし、次回の定期受診は出版会社が忙しい時期だったので職場からオンラインで実施することとした。本日（2月20日）、予定通りオンライン診療をこれから実施する。尚、これまでの診察は毎回、今回の医師役が対応している（今回で4回目）。

【既往歴】特記すべきことなし

【アレルギー歴】特記なし

【生活歴】飲酒：なし、喫煙：なし、仕事：出版会社で雑誌の編集をしている。

【家族歴】父：高血圧(当院通院中)、母：脂質異常症(当院通院中) 、両親と3人暮らし。

【身体所見】(初診時) 身長：160 ㎝、体重：54 ㎏、胸腹部診察：異常なし

【検査】(初診時) 血液：異常なし、心電図：異常なし、胸部 X 線：異常なし

この患者の診療を⾏ってください。時間は10分間です。

【患者役へのお願い】

1. **担当医に降圧目標について質問してください。**
2. **通常のオープンな質問では、空咳についての相談はしないでください。**

（他に何か相談しておきたいことは？何か話しそびれたことは？など、アクティブな問いかけがきたら「血圧とは全然関係ないことなので、言わなかったんですけど、最近咳が出るので、ついでに咳止めも出してもらえますか？」というセリフで切り出してください）

【身体所見】

血圧：124/72mmHg、脈拍：60/分（家庭血圧計で測定）、聴診：心音・呼吸音異常なし

【患者の想い】

血圧の治療が脳梗塞等の疾患を予防するために重要であることは理解している。これを機に、生活習慣にもしっかり取り組んで、薬もきちんと忘れずに飲み続けようと思っている。

仕事で帰りが遅くなり、夕食が遅くなることがあったので、そのような日は、仕事中に休憩をとり、職場で早めの夕食を摂るようにした。主治医のアドバイスを守って、汁物・煮物・漬物は控えて、酸味やだしを活用するようにしている。運動不足だったので、週末は 1 時間ぐらいウォーキングをするようになった。初診時から 4 か月程経過し、体重は 52（－2）㎏ と減少傾向である。両親は80 歳を超えて健在だが、いずれ両親の面倒をみるのは自分だし、両親を看取るまでは自分は健康でいる必要があると思っている。薬を始めてから、家庭血圧はいつも 上が 120 台前半、下が 70 台前半におさまっている。今回のオンライン診療では、先生に家庭血圧の目標値を確認してみようと思っている。前回の診察の少し前ぐらいから、軽い乾いた咳が出るようになったが、血圧とは関係ないだろうし、前回の診察の時には特に医師には相談しなかった。その後も、別に具合が悪いわけではなく、普通の生活には全く支障はない程度の軽い咳は続いていて、仕事の取材中にたまたま咳き込んで、コロナ禍なので白い目で見られたことがあった。今回のオンライン診療中に、ついでに相談できそうな雰囲気だったら、咳のことも話して、咳止めでも処方してもらおうと思っている。「エナラプリルの副作用が考えられる」と言われたら、「仕事で会う方に不快に思われたり、失礼に感じられるのは気がかりです。他の薬にしたら咳がなくなるなら助かるのですが…」と答えてください。

【評価のポイント】

①降圧目標や目的を明示し、適切な食事・運動療法・薬物療法の指導・管理ができる。

②患者の努力を称賛しつつ、その背景・言動力を抽出し、努力が継続するよう援助できる。

③慢性咳嗽のエピソードを聴取することができる。更に、慢性咳嗽の原因として重大な疾患を除外しつつ、アンギオテンシン変換酵素阻害薬の代表的副作用の可能性を想起し、適切な対応をとることができる。

2023/11

生活習慣病シナリオ②：**医師役**シート

**課題**

あなたはH町にある無床診療所の医師です。コロナ禍の収束後もオンライン診療に取り組んでいます。平日の午後（15時頃）、次の患者をオンラインで診療することになりました。

**患者：48歳　男性　佐藤太郎 さん**

**受診理由：アルコール性肝障害の定期診察（2回目）**

3か月前（X年8月1日）の職場健診で肝機能異常を指摘され、同年10月1日に当院を初診しました。血液検査を再検し、腹部エコーでは脂肪肝の所見のみでした。この日の外来は混んでおり、「お酒を控えるように」くらいのことしか言えませんでした。また、本人から「自分も忙しいので採血結果を1時間待っている時間がもったいない」という申し出があり、次回は事前（X年11月1日）に採血のみ行って、今日は検査結果説明を含めたオンライン診療を職場で受ける予定です。

【内服薬】なし

【既往歴】特記なし

【アレルギー歴】特記なし

【喫煙】なし

【職業】サラリーマン（営業担当）

【家族歴】特記なし

【家族構成】妻（42歳、主婦）、長男（高校3年）、次男（高校1年）の4人暮らし。

この患者の診療を⾏ってください。時間は10分間です。

|  | X年11月1日 | X年10月1日 | X年8月1日 |
| --- | --- | --- | --- |
| 白血球数 | 5100 | 4000 | 4300 |
| 赤血球数 | 555 | 534 | 530 |
| 血色素量 | 16.2 | 15.5 | 15.8 |
| ヘマトクリット | 48.4 | 46.2 | 46.0 |
| 血小板数 | 19.6 | 24.5 | 23.0 |
| MCV | 87 | 87 | 87 |
| MCH | 29.2 | 29.0 | 29.1 |
| MCHC | 33.5 | 33.5 | 33.5 |
| AST | H 75 | H 52 | H 44 |
| ALT | H 65 | H 49 | H 42 |
| γ-GTP | H 206 | H 113 | H 100 |
| 尿酸 | 6.9 | 6.4 | 6.8 |
| 血糖 | 99 | 82 | 80 |
| HbA1c | 6.1 | 5.6 | 5.2 |
| 中性脂肪 | H 395 | H 239 | H 210 |
| 総-cho | H 243 | 205 | H 260 |
| HDL-cho | L 39 | 44 | 55 |
| LDL-cho | 125 | 114 | H 163 |

2023/11

生活習慣病シナリオ②：**患者役**シート

**課題**

あなたはH町にある無床診療所の医師です。コロナ禍の収束後もオンライン診療に取り組んでいます。平日の午後（15時頃）、次の患者をオンラインで診療することになりました。

**患者：48歳　男性　佐藤太郎 さん**

**受診理由：アルコール性肝障害の定期診察（2回目）**

3か月前（X年8月1日）の職場健診で肝機能異常を指摘され、同年10月1日に当院を初診しました。血液検査を再検し、腹部エコーでは脂肪肝の所見のみでした。この日の外来は混んでおり、「お酒を控えるように」くらいのことしか言えませんでした。また、本人から「自分も忙しいので採血結果を1時間待っている時間がもったいない」という申し出があり、次回は事前（X年11月1日）に採血のみ行って、今日は検査結果説明を含めたオンライン診療を職場で受ける予定です。

【内服薬】なし

【既往歴】特記なし

【アレルギー歴】特記なし

【喫煙】なし

【職業】サラリーマン（営業担当）

【家族歴】特記なし

【家族構成】妻（42歳、主婦）、長男（高校3年）、次男（高校1年）の4人暮らし。

この患者の診療を⾏ってください。時間は10分間です。

|  | X年11月1日 | X年10月1日 | X年8月1日 |
| --- | --- | --- | --- |
| 白血球数 | 5100 | 4000 | 4300 |
| 赤血球数 | 555 | 534 | 530 |
| 血色素量 | 16.2 | 15.5 | 15.8 |
| ヘマトクリット | 48.4 | 46.2 | 46.0 |
| 血小板数 | 19.6 | 24.5 | 23.0 |
| MCV | 87 | 87 | 87 |
| MCH | 29.2 | 29.0 | 29.1 |
| MCHC | 33.5 | 33.5 | 33.5 |
| AST | H 75 | H 52 | H 44 |
| ALT | H 65 | H 49 | H 42 |
| γ-GTP | H 206 | H 113 | H 100 |
| 尿酸 | 6.9 | 6.4 | 6.8 |
| 血糖 | 99 | 82 | 80 |
| HbA1c | 6.1 | 5.6 | 5.2 |
| 中性脂肪 | H 395 | H 239 | H 210 |
| 総-cho | H 243 | 205 | H 260 |
| HDL-cho | L 39 | 44 | 55 |
| LDL-cho | 125 | 114 | H 163 |

【飲酒】ビール 1000ml＋焼酎水割り2杯/日、休肝日なし。

【食事】朝はごはんと味噌汁、昼食は妻の弁当、夕食も家で食べることが多い。

【運動】していない。時間があれば休日に散歩くらいはしてみようとは思う。

【睡眠】1時就寝、6時起床、熟睡感はない。ギリギリまで寝ていたいのに4～5時頃に目が覚めてしまい、日中眠くなって仕事に支障がある。

【コンテクスト】

仕事は営業で残業や休日出勤が多く、ノルマもあり、ストレスが大きい。家庭内でも長男が受験生でピリピリした雰囲気が常にある。晩酌が唯一のストレス発散になっている（晩酌以外のストレス解消法は現時点では特に思い当たらない）。妻が主婦で、息子たちは大学進学を希望しているので、自分が仕事を頑張ってしっかり稼がないといけないとは思っている。

【飲酒に関するかきかえ】

解釈：酒の量が多いから肝臓の数値が悪いことは理解している。

期待：なんとかしたいとは思っている。お酒を全く飲まない日（休肝日）は作りたくない。

感情：肝臓の病気になるのは怖い。

影響：症状はないし、日常生活に支障もない（睡眠との飲酒の関係は自覚していない）

【睡眠に関するかきかえ】

解釈：仕事のノルマや長男の受験がストレスになって睡眠が浅いのかな。

期待：6時起床のギリギリまで寝ていたい（睡眠時間を確保したい）。熟睡感がほしい。

感情：家族のために稼がないといけないので眠気のため仕事でミスしないか心配ではある。

影響：日中眠くなって仕事に支障が出ることもある。

【肝機能悪化および肝炎・肝硬変・肝癌などの説明を受けたら】

「肝臓の病気にはなりたくないのでなんとかしたい」と思う一方で「簡単にはやめられない」というスタンスで説明を聞いてください。

【検査の異常（肝臓）以外に飲酒が影響していると思うことはあるか聞かれたら】

「特にありません」と答えてください（睡眠と飲酒の関係は自覚していない）。

【飲酒の害に関する気づきを促しながら問題点を整理する】

睡眠と飲酒の関係（睡眠が浅くなることや早朝覚醒）について説明を受けたら、「お酒を飲むと寝つきが良くなるのでお酒は睡眠に良いと思っていました」、「かえって睡眠が浅くなったり、ムダに早起きする原因になるのですね」と気づきを得たリアクションをしてください。

【減酒を提案し、患者に合う方法をいっしょに考え、具体的な減酒目標を立てる】

気づきを得る前：「簡単には変えられない」というスタンスで答えてください。

気づきを得た後：「ビール 500ml＋焼酎水割り1杯/日でやってみます（全く飲まないのは難しい）と答えてください。

【CAGEの質問を受けたら】

すべて「いいえ」で答えてください。

＜参考＞　CAGE　アルコール依存症のスクリーニング

1. あなたは今までに、飲酒を減らさなければいけないと思ったことがありますか？

（Cut down）

2. あなたは今までに、飲酒を批判されて、腹が立ったり苛立ったことがありますか？

（Annoyed by criticism）

3. あなたは今までに、飲酒に後ろめたい気持ちや罪悪感を持ったことがありますか？

（Guilty feeling）

4. あなたは今までに、朝酒や迎え酒を飲んだことがありますか？

（Eye-opener）

【睡眠障害の鑑別にうつ病の2質問法が聞かれたら】

この1ヶ月間、気分が沈んだり、憂うつな気持ちになったりすることがよくありましたか？

この 1 ヶ月間、どうも物事に対して興味がわかない、あるいは心から楽しめない感じがよくありましたか？

→どちらも「いいえ」で答えてください。

【評価のポイント】

・患者の生活状況や考えを聴取し、飲酒の害について気づきを与えることができたか。

・具体的な減酒目標を立て、患者と相互意思決定することができたか。

・オンライン診療でも対面と同等の減酒支援（ブリーフインターベンション）ができたか。

参考：厚生労働省HPから


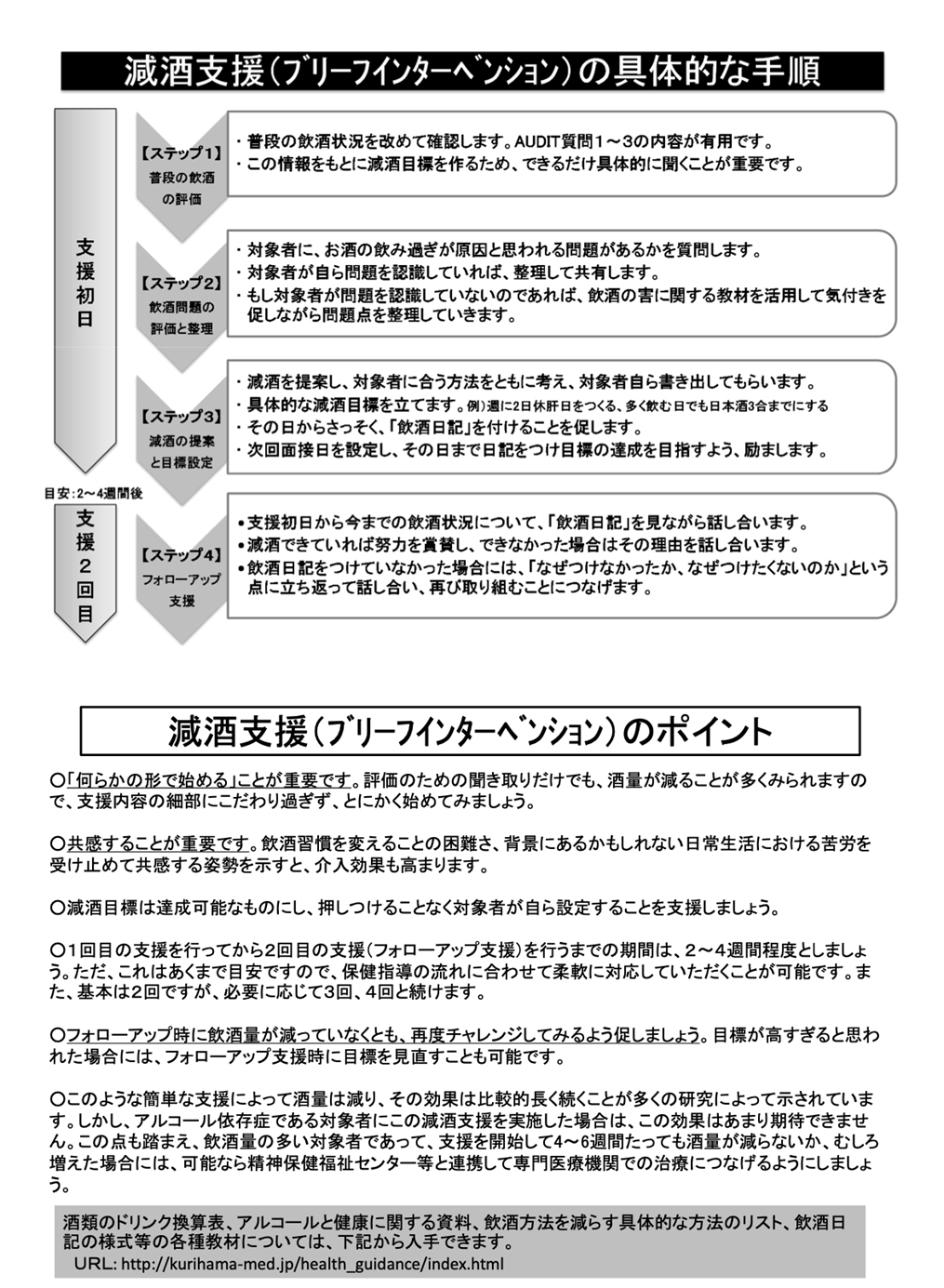


2023/12

シナリオ①：**医師役**シート

**課題**

あなたはH町にある無床診療所の医師です。コロナ禍の収束後もオンライン診療に取り組んでいます。オンラインの診察でも専用機器を用いて胸部の聴診は可能です。

平日の午後（15時頃）、次の患者をオンラインで診療することになりました。

**患児：10か月　男児　福島 大輝（ふくしま たいき）くん**

**主訴：皮疹**

【現病歴】来院前日の夕方、体幹部に皮疹が出ているのに母（父）親が気がついた。今朝になり、皮疹は体幹から四肢に広がってきたため心配になった。新型コロナウイルスやインフルエンザといった感染症が流行していて医療機関に行くことに不安があり、オンライン診療を希望した。鼻汁、咳嗽、咽頭痛、下痢など他の症状の訴えはない。

【既往歴】特記なし

【アレルギー歴】特記なし

【予防接種歴】：スケジュール通りに実施済み

【成長・発達】身長70㎝、体重8㎏、これまでの乳幼児健診では特に異常なし

【体温】36.8℃

【家族】両親と3人暮らし

【集団保育】：生後6か月から保育園

この患児の診療を⾏ってください。時間は10分間です。

2023/12

シナリオ①：**患者役**シート

**課題**

あなたはH町にある無床診療所の医師です。コロナ禍の収束後もオンライン診療に取り組んでいます。オンラインの診察でも専用機器を用いて胸部の聴診は可能です。

平日の午後（15時頃）、次の患者をオンラインで診療することになりました。

**患児：10か月　男児　福島 大輝（ふくしま たいき）くん**

**主訴：皮疹**

【現病歴】来院前日の夕方、体幹部に皮疹が出ているのに母（父）親が気がついた。今朝になり、皮疹は体幹から四肢に広がってきたため心配になった。新型コロナウイルスやインフルエンザといった感染症が流行していて医療機関に行くことに不安があり、オンライン診療を希望した。鼻汁、咳嗽、咽頭痛、下痢など他の症状の訴えはない。

【既往歴】特記なし

【アレルギー歴】特記なし

【予防接種歴】：スケジュール通りに実施済み

【成長・発達】身長70㎝、体重8㎏、これまでの乳幼児健診では特に異常なし

【体温】36.8℃

【家族】両親と3人暮らし

【集団保育】：生後6か月から保育園

この患児の診療を⾏ってください。時間は10分間です。

【全身状態】

・機嫌：少しいつもより機嫌が悪いかもしれない

・食欲：今朝いつもより食べたがらなかった

・睡眠：昨夜はいつも通りだった

【身体所見】

「皮疹を見せてください」と言われたら、以下の写真を画面共有で指導医が提示します。

- 特に指示がなければ１枚を提示します（やや暗く、インカメで解像度も悪め）。
- 医師から「ライトをつけてor部屋をできるだけ明るくして」「アウトカメラで映して」「近寄って映して」などと指示されたら、２枚目、３枚目の鮮明な画像を提示します。

体幹、四肢に紅色丘疹が散在、特に集簇傾向はない。背部の皮疹の一部は水疱化している。 母親は気づいていないが、指摘して観察をしてもらうと、頭皮や肛門部にも同様の皮疹がある。口腔内の観察は難しいが、口の中を気にする様子が今朝あったとのこと。

【母（父）親のかきかえ】

解釈：保育園で2週間前に仲良く遊んでいた子が、先週はずっとお休みだったらしい。詳細は聞いていないが、人からうつる病気なのか。その他、思い当たることはない。

期待：診断してほしい、この後どう対処してあげたらいいのか教えてほしい。

感情：皮疹が昨日より広がってきているので、悪化しているのではと心配している。

影響：自分も夫（妻）も働いているので、保育園を休ませる必要がある場合、どのくらいの期間休む必要があるのか。

★医師から自主的に情報提供がなければ、次のことを確認してください

「登園許可証はいつもらいに行けばいいですか？」

「親が気を付けるべきことはありますか？」

【父母の健康状態】

父：基礎疾患なし　水痘罹患歴あり

母：基礎疾患なし　妊娠なし　水痘抗体あり（妊娠時に確認）

現在妊娠している可能性は１００％なし

【評価のポイント】

・オンライン診療では、患者へ診療環境やカメラ操作を指示して、より精度の高い視診を心がけることができる

・典型的な経過の水痘を診断し、治療方針を説明することができる。

・父母のかきかえを探り、心配や不安を和らげることができる。

・父母の水痘予防接種歴および罹患歴、母の妊娠の有無を確認し、適切に予防策を相談できる

・登園の停止も含めた療養指導や周囲への適切な感染予防策を講じることができる。

**付録＜水痘の関連学習＞**

【疫学】

・水痘は水痘帯状疱疹ウイルス（VZV）の初感染による病態で、冬期、春期に多い。

・VZVは感染者からの飛沫やエアロゾルによる空気感染、または水疱との接触感染により伝播する。

・水痘感染者は通常、皮疹が現れる2日前から、すべての皮疹が痂皮化するまで感染性を持つ→ 全ての皮疹が痂皮化するまで出席停止（幼児～学童であれば約 1 週間程度）

【水痘ワクチン】

・水痘ワクチン（生ワクチン）は 2014 年（平成 26 年）10 月 1 日に定期接種化された

（2 回接種：1 回目を 1 歳、2 回目を 1 回目の 6 か月～12 か月後に接種するのが標準的）。

・水痘ワクチンの 1 回接種で水痘の罹患を 80～85%程度、重症化をほぼ 100%防ぐことができる。

・ワクチンを２回接種することで、1 回接種と比べて長期にわたり患者数を減らすことができる。

【病理】

・水痘は呼吸器感染で伝播する。VZV は飛沫やエアロゾルにより口腔咽頭や上気道、結膜の上皮細胞やリンパ球に感染する。VZV は感染したリンパ球により全身に運ばれ、血管外に移動して皮膚に侵入し、皮疹を形成する。

・皮膚病変は紅色丘疹～水疱だが、炎症細胞が浸潤すると膿性になる。その後皮疹は崩壊して痂皮を形成し、瘢痕を残さずに治癒する。

・VZV は初感染の際、脳神経や脊髄後根の神経節の神経細胞内に潜伏感染する。VZV に特異的な細胞性免疫が衰えると、VZV は神経節で再活性化し、軸索を下行して上皮細胞内で増殖し、帯状疱疹を発症する。

【臨床経過】

・水痘の潜伏期間は約 14 日間（10～21 日間）。

・水痘は通常、発熱、倦怠感で発症し、1～2 日後に全身性の皮疹が現れる。ただし水痘患者の約 3 割は発熱しない。

・皮疹は最初に紅色丘疹が出現し、水疱、膿疱、痂皮の順に進行する。3 日程度皮疹が新生するため、各ステージの皮疹が同時に存在するのが特徴。

・皮疹は躯幹、顔面に多い。粘膜病変がみられることもある（口内炎、歯肉炎）。

・頭部有髪部位や肛門周囲の皮疹は他疾患で認めることが少なく、診断的意義が高い。

【合併症】

・水痘の最も頻度の高い合併症は、皮膚病変への細菌の重複感染（A 群連鎖球菌や黄色ブドウ球菌による蜂窩織炎、菌血症、壊死性筋膜炎など）。

・他の合併症として小脳失調（歩行障害、構音障害等を呈する）、ウイルス性肺炎、肝炎、血小板減少症などがある。

・稀な合併症としてウイルス性髄膜炎、脳炎、血管障害（脳卒中として発症）等がある。

【診断】

・水痘、帯状疱疹ともに臨床診断。水痘・帯状疱疹患者との接触歴（約 2 週間前）、段階の異なる皮疹が躯幹、顔面を中心に播種性に多発散在していれば、ほぼ水痘と診断できる。

・VZV 特異的 IgM の上昇には発疹出現後数日、IgG の上昇には 1 週間程度要するため、水痘の診断における血清学的検査の意義は小さい。

・鑑別診断は膿痂疹、エンテロウイルス感染症（手足口病など）、単純ヘルペス、Stevens-Johnson 症候群等。

【治療】

・アシクロビル/バラシクロビル内服により水痘の有熱期間は約 1 日短縮され、出現する皮疹の個数も少なくなる。内服による副作用もほとんど見られないが、水痘合併症の頻度や、水疱痂皮化までの期間は変わらない。

・小児にはバルトレックス顆粒 5 日間内服（添付文書上は、皮疹出現から 2 日以内に投与を開始することが望ましい）。成人の水痘は 5～7 日間の内服。

・水痘の外用薬としては亜鉛華単軟膏が勧められる（消炎、分泌物の吸収、創部の保護の他、皮疹を被覆して VZV の拡散を抑える効果が期待できる）。

※海外の文献ではアシクロビル/バラシクロビル内服の適応は 12 歳以上、および基礎疾患を持った患児等に限られ、健康な幼児・学童での有益性に関しては議論がある。

【感染予防、曝露後の水痘発症予防】

・水痘を発症した場合、皮疹がすべて痂皮化するまでは、周囲への空気感染、接触感染の予防を行う。

・水痘ウイルスへの曝露が 3 日以内である場合、水痘ワクチン緊急接種で水痘発症を 70～90％予防できる。

・アシクロビル/バラシクロビル内服でも水痘発症を 80～85%予防できる（曝露後 8 日目から、7 日間投与（保険適用外））。

・水痘ワクチン接種や罹患歴がない妊婦には、理想的には水痘抗体検査を実施の上、暴露後１０日以内にガンマグロブリンを投与を検討する（適宜産科へコンサルト。保険適用外）。また、妊娠中に水痘を発症した場合はアシクロビル/バラシクロビルで加療する。

【水痘感受性者の曝露後予防（免疫不全のない場合）】

曝露後 3～4 日以内

・生ワクチンの禁忌がなければ、水痘ワクチン緊急接種を検討。

・生ワクチンの禁忌があれば、アシクロビル/バラシクロビル内服を検討（曝露後 8 日目から、7 日間投与）

曝露後 3～4 日以降

・アシクロビル/バラシクロビル内服を検討（曝露後 8 日目から、7 日間投与）

※曝露後予防としてのアシクロビル/バラシクロビル内服は保険適用外。

【参考文献】

1. 厚生労働省 健康局 結核感染症課 予防接種室 第 3 回予防接種基本方針部会. 水痘ワクチンの接種対象者及び接種方法について. 平成 25 年７月 10 日.

<https://www.mhlw.go.jp/file/05-Shingikai-10601000-Daijinkanboukouseikagakuka-Kouseikagakuka/0000034764_2.pdf>

1. Cohen J. Varicella-zoster virus (chickenpox, shingles). In Goldman L, Schafer AI （eds）: Goldman-Cecil Medicine 26th edition. Philadelphia: Elsevier 2020; 2192-2195.
2. Klassen TP, Hartling L. Acyclovir for treating varicella in otherwise healthy children and adolescents. Cochrane Database of Systematic Reviews 2005, Issue 4. Art. No.: CD002980. DOI: 10.1002/14651858.CD002980.pub3. Accessed 28 January 2021.
3. 森内浩幸. 水痘を中心としたウイルス感染症の院内感染制御. 小児感染免疫 Vol. 22 No.2 p181-186, 2010
4. Varicella-zoster virus infection in pregnancy. Up To Date (閲覧：2024.11.27.)
5. 日本産婦人科学会, 日本産婦人科医会. 妊産褥婦の水痘感染については？. 産婦人科診療ガイドライン―産科編 2020


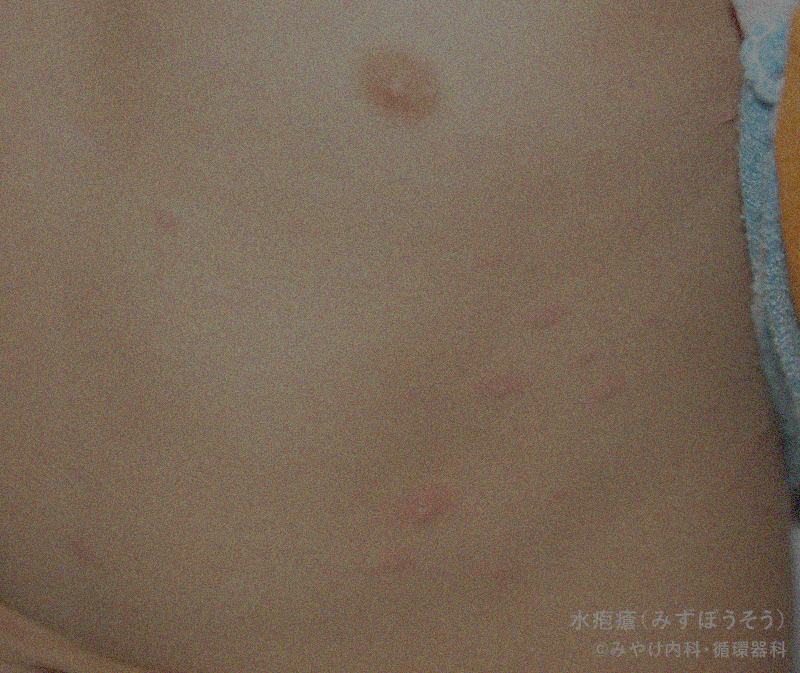
皮疹の画像①

皮疹の画像②


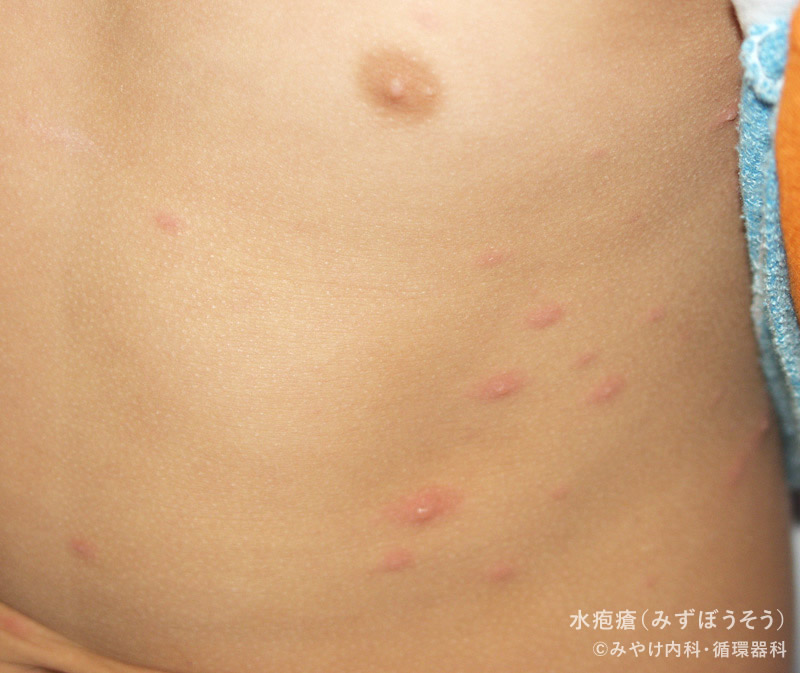


皮疹の画像③


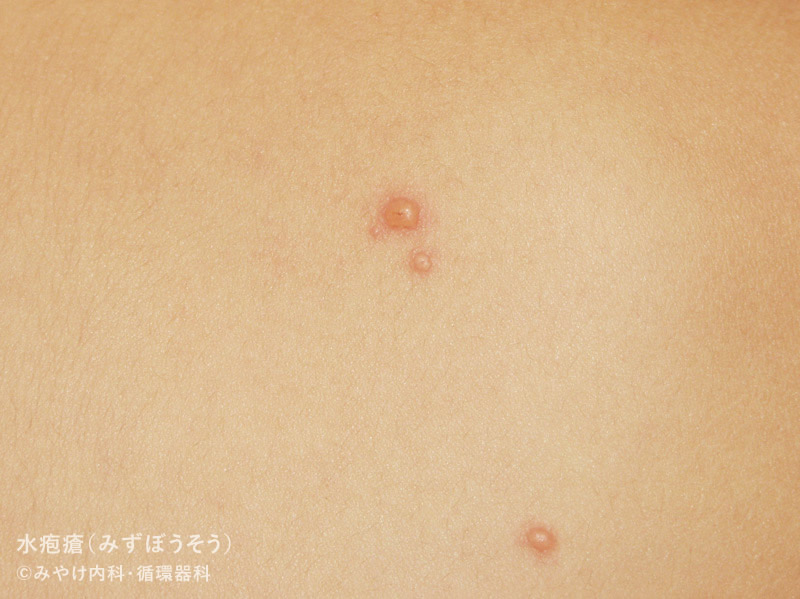


2023/12

シナリオ②：**医師役**シート

**課題**

あなたはH町にある無床診療所の医師です。コロナ禍の収束後もオンライン診療に取り組んでいます。オンラインの診察でも専用機器を用いて胸部の聴診は可能です。

平日の午後（15時頃）、次の患者をオンラインで診療することになりました。

**患児：1歳6か月　男児　花見 潤（はなみ じゅん）くん**

**主訴：鼻汁**

【現病歴】2日前に鼻汁、咳を主訴に母親（父親）に伴われ当院を受診し、あなたが担当しました。その時は発症初日で症状は軽く全身状態良好であり、急性上気道炎と診断、カルボシステイン内服のみ処方した。その後、鼻水がややひどくなってきたようで、夜苦しそうにしていた。診療所に連れていくほどではないかなと思いながらも、今夜も寝苦しいとかわいそうと思い、保育園から帰宅後１７時過ぎに少し相談したくてオンライン診療を希望した。

【既往歴】特記なし

【アレルギー歴】特記なし

【予防接種歴】：スケジュール通りに実施済み

【成長・発達】身長80㎝、体重10㎏、これまでの乳幼児健診では特に異常なし

【体温】36.8℃

【家族】両親と3人暮らし

【集団保育】：生後6か月から保育園

この患児の診療を⾏ってください。時間は10分間です。

2023/12

シナリオ②：**患者役**シート

**課題**

あなたはH町にある無床診療所の医師です。コロナ禍の収束後もオンライン診療に取り組んでいます。オンラインの診察でも専用機器を用いて胸部の聴診は可能です。

平日の午後（15時頃）、次の患者をオンラインで診療することになりました。

**患児：1歳6か月　男児　花見 潤（はなみ じゅん）くん**

**主訴：鼻汁**

【現病歴】2日前に鼻汁、咳を主訴に母親（父親）に伴われ当院を受診し、あなたが担当しました。その時は発症初日で症状は軽く全身状態良好であり、急性上気道炎と診断、カルボシステイン内服のみ処方した。その後、鼻水がややひどくなってきたようで、夜苦しそうにしていた。診療所に連れていくほどではないかなと思いながらも、今夜も寝苦しいとかわいそうと思い、保育園から帰宅後１７時過ぎに少し相談したくてオンライン診療を希望した。

【既往歴】特記なし

【アレルギー歴】特記なし

【予防接種歴】：スケジュール通りに実施済み

【成長・発達】身長80㎝、体重10㎏、これまでの乳幼児健診では特に異常なし

【体温】36.8℃

【家族】両親と3人暮らし

【集団保育】：生後6か月から保育園

この患児の診療を⾏ってください。時間は10分間です。

～母親（父親）の雰囲気～　不安や不満をあらわにする様子はない。

**「その後いかがですか？」といった医師役の最初の質問に対してのセリフ**

先日診てもらったばかりなのですが、鼻水がややひどくなってきたようで、夜苦しそうにしていたんです。今日保育園でも元気はあったみたいなのですが、今夜もまた寝苦しくなってしまうんじゃないか心配で診てもらおうと思いました。

**詳細な病歴**

2日前、起床時から鼻水がグズグズしており、熱を出す前に早めに受診したほうが良いだろうと考え当院を受診した。先生の診察や説明も丁寧で安心して帰宅した。熱は37.2℃が最高で、元気も食欲も十分ある。昨日も保育園に行けている。昨夜は布団に入ってから鼻が苦しそうで、咳をして夜中に何度も起きた。ゼーゼーしてはいないが、口呼吸で苦しそうだった。本日起きてからは元気そうだったので保育園に登園した。お迎えの際に保育園の先生は「鼻水は多いけど、元気に過ごしていました」と言われた。

鼻水は出てるものはティッシュで拭いたり、綿棒で取れるものは取っている。

鼻の奥にたまった鼻水をどうしていいかわからない。鼻吸い器は使用したことない。

縦に抱っこすると寝つくが、また横にすると苦しがり、グズグズしている。

食欲は普段より落ちているが食べれている。日中は普段通り遊べており、ぐったりはしていない。

**身体所見**

鼻閉はあるが呼吸は穏やか、機嫌も悪くない。鼻孔には鼻水べったり、漿液性鼻汁。

胸部聴診で異常を認めない。

**母親（父親）のかきかえ**

解釈：保育園に通うようになりたびたび風邪をひくが、しょうがないと思っている。今回も肺炎など重病を心配しているわけではない。

期待：寝苦しさを和らげてほしい。鼻水をどうにかしてほしい（もっとよく効く薬がほしい。抗生剤とかもらうと手っとり早く治るのだろうかと期待）。

感情：寝不足で自分も今朝から喉が少し痛く、ややだるい。

影響：自分も寝れない。風邪移ってしまった（まだ仕事はできそう）

★一般的な対症療法の指導のみの場合、「先生、もう少し何か鼻水なくす方法とかないでしょうか。毎晩苦しそうなので親の自分たちも寝不足で・・・」と訴えてください。

　→医師の共感的な態度や丁寧な風邪の説明やホームケアの指導があれば、納得がいった反応を示してください。

**評価のポイント**

・鼻汁症状への薬物的・非薬物的なホームケアを指導できる。

・オンライン診療では、手軽さ故に「コンビニ受診」の傾向のある患者に遭遇しうる。医療者自身の陰性感情に注意を払いながら、ヘルスリテラシーを高める働きかけができる。

・母親（父親）のかきかえを探り、本日の受診理由を理解し、親の苦悩も受容できている。

・どのような場合に再診（対面診療）が必要か、セーフティーネットを張ることができる。

2024/1

シナリオ①：**医師役**シート

**課題**

あなたはH町にある無床診療所の医師です。コロナ禍の収束後もオンライン診療に取り組んでいます。平日の午後（15時頃）、次の患者をオンラインで診療することになりました（この地域でインフルエンザが流行しており、患者が来院をためらったためです）。1回目と2回目に対面で実施した診察・検査により以下のことが分かっています(本日は3回目、検査結果は全て患者に説明済です)。患者は自宅の個室からオンライン診療を受けます。

**患者：25歳　女性　出須久 和久(ですく わく) さん**

**主訴：頭痛、腹部の違和感**

3か月前から頭痛、腹部不快感(重い感じ)が出現した。腹痛、嘔気・嘔吐、排便の変化はない。仕事が忙しいときに多い傾向があり、この症状のため、月に数回は仕事を休むこともある。内科などの医療機関を受診し、投薬などを受けたりしたが効果が乏しかった。

【既往歴】特記すべきことなし

【内服薬】なし（サプリメントもなし）

【アレルギー歴】特記なし

【生活歴】飲酒：機会飲酒、喫煙：なし、仕事：事務職（自動車部品メーカーの経理）

【学歴】国立大学の経済学部卒

・器質的疾患精査を行った結果は以下の通り

身体診察：特記すべき異常は見当たらず、中肉中背、体重変化はない。

血液検査・尿検査で異常なし（妊娠もなし）

腹部超音波、上部消化管検査も異常なし

前回（2回目）の診察時にうつ病を疑い、評価スケールPHQ-9を実施したところ、以下の通り●/30点で、中等度うつ病を疑った。

Qここ2週間で 次のような問題にどのくらい頻繁に悩まされているか?

1物事に対してほとんど興味がない。または楽しめない →半分以上

2気分が落ちこむ、憂鬱になる、または絶望的な気持ちになる →半分以上

3寝つきが悪い。途中で目が覚める、または逆に眠りすぎる →半分以上

4疲れた感じがする、または気力がない →半分以上

5あまり食欲がない、または食べ過ぎる →週に数日

6自分はダメな人間だ、人生の敗北者だと気に病む。 →なし

7新聞を読む、またはテレビを見ることに集中することが難しい →半分以上

8他人が気付くぐらいに動きや話し方が遅くなる。あるいは反対にそわそわして落ち着かない→週に数日

9死んだ方がましだ、あるいは自分をなんらかの方法で傷つけようと思ったことがある

→なし

10上記の症状によって仕事をしたり、家事をすることがどれくらい困難か? →やや困難

前回の診療の時点で「うつ病が疑われる」と患者に伝えたが、「う～ん・・・」と黙り込んでしまい、1週間後の本日に診断と治療方針について話し合うことにした。

この患者の診療を⾏ってください。時間は10分間です。

診療中に、リーフレット「うつ病の身近な情報」（添付）を適宜画面共有しながら活用しても構いません。

2024/1

シナリオ①：**患者役**シート

**課題**

あなたはH町にある無床診療所の医師です。コロナ禍の収束後もオンライン診療に取り組んでいます。平日の午後（15時頃）、次の患者をオンラインで診療することになりました（この地域でインフルエンザが流行しており、患者が来院をためらったためです）。1回目と2回目に対面で実施した診察・検査により以下のことが分かっています(本日は3回目、検査結果は全て患者に説明済です)。患者は自宅の個室からオンライン診療を受けます。

**患者：25歳　女性　出須久 和久(ですく わく) さん**

**主訴：頭痛、腹部の違和感**

3か月前から頭痛、腹部不快感(重い感じ)が出現した。腹痛、嘔気・嘔吐、排便の変化はない。仕事が忙しいときに多い傾向があり、この症状のため、月に数回は仕事を休むこともある。内科などの医療機関を受診し、投薬などを受けたりしたが効果が乏しかった。

【既往歴】特記すべきことなし

【内服薬】なし（サプリメントもなし）

【アレルギー歴】特記なし

【生活歴】飲酒：機会飲酒、喫煙：なし、仕事：事務職（自動車部品メーカーの経理）

【学歴】国立大学の経済学部卒

・器質的疾患精査を行った結果は以下の通り

身体診察：特記すべき異常は見当たらず、中肉中背、体重変化はない。

血液検査・尿検査で異常なし（妊娠もなし）

腹部超音波、上部消化管検査も異常なし

前回（2回目）の診察時にうつ病を疑い、評価スケールPHQ-9を実施したところ、以下の通り●/30点で、中等度うつ病を疑った。

Qここ2週間で 次のような問題にどのくらい頻繁に悩まされているか?

1物事に対してほとんど興味がない。または楽しめない →半分以上

2気分が落ちこむ、憂鬱になる、または絶望的な気持ちになる →半分以上

3寝つきが悪い。途中で目が覚める、または逆に眠りすぎる →半分以上

4疲れた感じがする、または気力がない →半分以上

5あまり食欲がない、または食べ過ぎる →週に数日

6自分はダメな人間だ、人生の敗北者だと気に病む。 →なし

7新聞を読む、またはテレビを見ることに集中することが難しい →半分以上

8他人が気付くぐらいに動きや話し方が遅くなる。あるいは反対にそわそわして落ち着かない→週に数日

9死んだ方がましだ、あるいは自分をなんらかの方法で傷つけようと思ったことがある

→なし

10上記の症状によって仕事をしたり、家事をすることがどれくらい困難か? →やや困難

前回の診療の時点で「うつ病が疑われる」と患者に伝えたが、「う～ん・・・」と黙り込んでしまい、1週間後の本日に診断と治療方針について話し合うことにした。

この患者の診療を⾏ってください。時間は10分間です。

診療中に、リーフレット「うつ病の身近な情報」（添付）を適宜画面共有しながら活用しても構いません。

-------------------------------------------------------------------------------------------------------

診断：うつ病(中等度、いまのところ躁エピソードはない)

〜作問の狙い〜

・うつ病の病状説明がしっかりできるかどうか?

・治療方針について共通の理解のもと意思決定できるかどうか?

・オンラインでの会話の難しさをクリアできるか？

（ぼそぼそと話す→なるべく大きな声で話すように促すなどできるか）

〜模擬患者設定〜

・うつ病なので、暗い表情、どんよりした雰囲気をだしてください。

・最初、なるべくボソボソと話して聞こえにくくしてください。

・話し方もゆっくりで、集中力もないので、似たようなセリフ(例えば、私がうつ病なんて…、やつ〈上司〉のせいで…)をなんどかボヤいて(反復思考して)、”心ここにあらず”な人を演じてください。医師役の配慮の声かけを引き出すつもりで！

・頭痛、腹部の違和感の症状は、3 ヶ月前から改善なく続いている。

・これまで何か所か医療機関を受診して薬(鎮痛剤、胃薬)ももらったが、効果なし。

・上司との対人関係に問題を抱えている：折り合いが悪い(例：新しく担当することになった仕事がわからないことだらけだが、上司が忙しそうかつ怒りっぽい。なかなか聞けなくて悶々とする)

・うつ病(精神疾患)であることを他人に知られたくない

・生活習慣はかなり乱れている(土日は昼まで寝ている、夜はおそくまでTiKTok等を見ている、運動などは一切できていない)。実家からそう遠くないところに一人暮らし。

・父：公認会計士、母：専業主婦、兄弟はいない。本当は父のような公認会計士になりたかったが、結局なれず、コンプレックスあり。

・趣味：TikTokをみる。ネタ系や踊ってみた系をダラダラと見ている。気づくと2～3時間、時間が過ぎている。

〜模擬患者の演技どころ〜

うつ病に関する偏見を持っている患者を演じてください。

・うつ病にかかるのは 精神的に弱い人間だ(私は弱い人間ではない！)

・私の頭痛や腹部の違和感は身体の問題だ。気持ちの問題ではない。

・うつ病と診断されたら、精神科にいかなければならない(精神科に行くと、頭のおかしい、弱い人間だと思われるので、絶対に精神科には行きたくない)

・症状が良くなるなら薬は飲んでもいいが、副作用が心配、やめられなくなるのが心配。

〜マネジメントに関しての設定〜

・できれば(うつ病の)治療をここで継続してほしい。

・どうしても紹介を勧められたら、(精神科でも心療内科でも)しぶしぶ同意はする。

〜”かきかえ”にすると〜

解釈：具体的に心配する病気はないが、身体になにか病気があるのではないか？

期待：頭痛やおなかの重い感じの原因は何？症状を良くして仕事に取り組みたい。

感情：どうしていいかわからない。不安。また上司に怒られるかも。ゆううつ。自分はデキる人間のはずなのに(プライドと現状のギャップに葛藤)。

影響：仕事を何度か休んでしまった。生活習慣も乱れてきた。

【評価のポイント】

・うつ病と認識できているか（躁エピソードの有無、不安障害の評価、幻覚幻聴の有無等）

・患者の偏見を聞き出せているか、偏見に適切に答えられているか。

・”心ここにあらず”な様子(キュー)に気がついているか。

・適宜理解度を確認し、話を進められているか（患者に理解した内容を言ってもらう等)。

・理解を促すために、リーフレットなど患者向け情報をオンラインでも有効活用できるか。

・患者の希望を考慮したうえで、治療方針を決定できているか。

・対面診療を含むセーフティーネットやフォローアップを説明・約束できているか。

<参考資料> うつ病の正しい説明

・うつ病はだれでも罹りうる(日本の生涯有病率 7.5%:14人に一人 厚労省 Q&A より)、心の弱い人だけがなるものではない。

・気分の落ちこみや疲れた感じがあり、以前のように人生を楽しむことができなくなっている状態。これまでできていた生活や仕事が困難になる場合もあり。

・うつ病の原因はまだ不明な点も多く、さまざまな要因が関わっているとされる。環境の

変化に伴う精神的なストレスなどにより脳の働きに不調をきたすことが知られている。

・気持ちと身体症状には関係がある。

・治療法があり、適切な治療で多くの方が改善する(しかし5-10%の方が慢性化する)

・軽度〜中等度のうつ病であれば、適切に生活習慣を改善・整えたりして、必要に応じて

　薬も併用すれば(必ずしも精神科に通院しなくても)改善する場合がある。

・うつ病をほうったままにして無理を続けると、重症になり、社会的な生活が困難になる

　ほか、最悪死ぬ(自殺する)場合もある。

　情報源の1つ：こころの健康情報のページ(パンフレット等) - 福島県

<https://www.pref.fukushima.lg.jp/sec/21840a/mhealth-1.html>

（添付したリーフレットもここにある）

【治療法について】

・生活習慣の改善(規則的な生活、睡眠衛生の改善、運動、社会的活動の維持・向上等

・必要ならストレスから離れるために休む場合もある。

・カウンセリング

・薬(抗うつ薬)：副作用は消化器症状が多い(まれに傾眠や性的機能低下)。最初の1〜2週

は副作用がつらい場合があるが、3〜4週程して気分の改善がみられる。気分が安定して6か月程すれば終了できる場合もある。

2024/1

メンタルヘルスシナリオ②：**医師役**シート

**課題**

あなたはH町にある無床診療所の医師です。コロナ禍の収束後もオンライン診療に取り組んでいます。平日の午後（15時頃）、次の患者をオンラインで診療することになりました。

（この地域でインフルエンザが流行しており、患者が来院をためらったためです）。1回目に対面で実施した診察・検査により以下のことが分かっています(本日は2回目、検査結果は全て患者に説明済です)。患者は自宅の個室からオンライン診療を受けます。

**患者：22歳　女性　橋本 月乃（はしもと つきの）さん**

**主訴：ぼーっとする**

もともと数か月の単位で気分の浮き沈みはある。生理の前に落ち込むことが多い。基本的にはハイテンションに過ごすことが多く、友人にも明るいキャラだと思われている。今年7月頃に生理痛がひどくて落ち込んだ時期もあったが秋頃は調子よく過ごしてきて、また寒くなってきてから調子が悪い。

【既往歴】特記すべきことなし

【内服薬】なし（サプリメントもなし）

【アレルギー歴】特記なし

【生活歴】飲酒：機会飲酒、喫煙：なし、趣味：友人と好きなバンドのライブにいくこと。

【社会歴】高校卒業後、2 度転職して現在工場の従業員として 2 年間勤務している。

【家族】両親と３人暮らし。3 つ上の姉がいるが結婚し、親元を離れて生活している。

・器質的疾患精査を行った結果は以下の通り

身体診察：特記すべき異常は見当たらず、中肉中背、体重変化はない。

血液検査：血算 CRP 肝機能 腎機能 電解質(Na K Cl Ca) 血糖 HbA1c フェリチン 甲状腺機能 副腎機能(ACTH コルチゾール) 全て異常なし。

尿検査：異常なし (妊娠もなし)

この患者の診療を⾏ってください。時間は10分間です。

2024/1

メンタルヘルスシナリオ②：**患者役**シート

**課題**

あなたはH町にある無床診療所の医師です。コロナ禍の収束後もオンライン診療に取り組んでいます。平日の午後（15時頃）、次の患者をオンラインで診療することになりました。

（この地域でインフルエンザが流行しており、患者が来院をためらったためです）。1回目に対面で実施した診察・検査により以下のことが分かっています(本日は2回目、検査結果は全て患者に説明済です)。患者は自宅の個室からオンライン診療を受けます。

**患者：22歳　女性　橋本 月乃（はしもと つきの）さん**

**主訴：ぼーっとする**

もともと数か月の単位で気分の浮き沈みはある。生理の前に落ち込むことが多い。基本的にはハイテンションに過ごすことが多く、友人にも明るいキャラだと思われている。今年7月頃に生理痛がひどくて落ち込んだ時期もあったが秋頃は調子よく過ごしてきて、また寒くなってきてから調子が悪い。

【既往歴】特記すべきことなし

【内服薬】なし（サプリメントもなし）

【アレルギー歴】特記なし

【生活歴】飲酒：機会飲酒、喫煙：なし、趣味：友人と好きなバンドのライブにいくこと。

【社会歴】高校卒業後、2 度転職して現在工場の従業員として 2 年間勤務している。

【家族】両親と３人暮らし。3 つ上の姉がいるが結婚し、親元を離れて生活している。

・器質的疾患精査を行った結果は以下の通り

身体診察：特記すべき異常は見当たらず、中肉中背、体重変化はない。

血液検査：血算 CRP 肝機能 腎機能 電解質(Na K Cl Ca) 血糖 HbA1c フェリチン 甲状腺機能 副腎機能(ACTH コルチゾール) 全て異常なし。

尿検査：異常なし (妊娠もなし)

この患者の診療を⾏ってください。時間は10分間です。

生活の変化で負担がかかったことを契機に抑うつの状態が悪化したが、実は双極性障害の可能性がある症例

入室時の様子：覇気がなく、声も暗め、話すスピードは普通

**＊医師が声をかけるまで画面はＯＦＦのまま**

**＊たまに猫が鳴き、そちらに席を外すことがある**

最初の語り出し：秋は調子よく過ごせていて、寒くなってきた時から、ぼーっとしたり、だるさがひどくなってきています。もともと季節によって体調の波があるんですけどね。

最近ではぼーっとして車の運転中に信号無視してしまったり、右折レーンで直進してしまったりしたので、これは危ないと思っていました。

以下は、医師役の問いかけに応じて答えてください。

【症状の OPQRST】

Onset：今年の 11 月はじめ頃からです。きっかけは…子猫のことかもしれないです。

Provocative/Palliative：家に帰ってくると憂鬱な気分になり、だるさも強く感じます。

Quantity/Quality：ぼーっとして、人の話が入ってこないというか、「うん…」と聞くんですけど、理解していないような、そんな感覚です。運転中もぼーっとしています。

Region/Radiation：耳がふさがるような感覚もあります。聞こえにくいような、そんな感覚です。あと、ちょっとドキドキするような、焦るような感じもします。

Symptoms：ふと、涙が出ることが多くなっています。今も先生と話していて、なんか涙が出そうになっています。夜眠れないことが多くなっています。

Time course/Treatment：だるさやぼーっとする感じは段々ひどくなっているような気がします。

【かきかえ】

解釈：子猫のことではストレスになっていると思います。

期待：この怠さは良くしてほしい。もとのように元気になりたいです。

感情：疲れが抜けなくて自分ではどうしたらいいのか上手く考えられない。明るく友人と遊んでいるのがいつもですが、今のまま落ち込みがひどかったら、友人たちにも呆れられるんじゃないかと思います。いっそ消えてしまったら楽になるのかな…。

影響：仕事や運転など日常生活に支障が出てしまっています。

【家族】

両親ともに精神科通院歴はない。父親は別の工場で働いている。患者とはあまり会話せず

家の中で影が薄い。母親の気分の浮き沈みはしょっちゅうある。母親が捨て猫を預かるボ

ランティアをしているが、1 か月前から生まれたての子猫を世話している。1時間おきの

ミルクを飲ませたりしてもなかなか体重も増えなくて心配している。子猫を預かったのは

初めてで、母親も参っている様子。母親は機嫌が悪く、自分が出かけようとすると難色を

示したりするのでストレスが溜まっている。本日、母親は子猫を患者に預けて出かけてお

り、夜まで帰ってこない予定とのこと。

【仕事】

疲れのせいかぼーっとすることが多く、前はテキパキと仕事ができていたのに仕事中もぼーっとしてしまう。

【その他】

・睡眠：次の日の仕事のことを考えると気分が落ちて、眠つけない日が増えています。

・食欲：だるさがひどくて食欲もあまりありません。

・２質問法： 抑うつ気分―あり、涙が出てくる感じ。興味の減退―あり、今は友人とライブに行きたい気持ちとか沸いてこない。

・躁のエピソード：調子がかなりいい時があって、カラオケでオールしたり、ライブで大騒ぎしたり、誰に文句言われたり悪口言われても無敵の状態っていうときが定期的にあります。

・自殺企図：死のうって、高校の時に思っていた時もあって、その時は死ぬ道具とか準備した時もありましたが、今はそういう風には考えないようにしようって思っています。でも、全部投げ出して、もう「消えてしまいたい」って思うことはあります。ここ最近そう思う頻度が増えていると思います。

【評価のポイント】

・オンラインで画面がうつらない、子猫の世話に席を立ってしまうといったトラブルにもオンライン診療の環境を整えるように対応する。

・抑うつ傾向にある患者に支持的な態度でコミュニケーションを図れているか。

・うつ病の病歴聴取、躁うつ病の可能性があるエピソードも聞き出せているか。

・精神科への紹介を判断できているか。

・病気の経験やコンテクストを探り、本人と診療方針について話し合いを持てているか。

患者さんへの病状説明の資材の一つ

「双極性障害（躁うつ病）と つきあうために」日本うつ病学会 双極性障害委員会

<https://www.secretariat.ne.jp/jsmd/gakkai/shiryo/data/bd_kaisetsu_ver9-20180730.pdf>

2024/2

シナリオ①：**医師役**シート

**課題**

あなたはH町にある無床診療所の医師です。診療所では外来診療の他に24時間体制の在宅診療を行っています。また、コロナ禍の収束後もオンライン診療に取り組んでいます。平日の午後（15時頃）、次の患者をD to P with N（訪問看護師が訪問した状態でのオンライン診療）で診療することになりました。患者宅は当院から片道40分かかるため、オンライン診療も織り交ぜながらの診療方針に本人・家族も同意しています。

**患者：82歳　女性　郡山 和美(こおりやま かずみ) さん**

高血圧で2か月に１回当院に通院していました。X-1年11月に外出中に転倒し、近隣のB総合病院に救急搬送、大腿骨頸部骨折の診断で入院しました。X年1月にリハビリを終えて自宅退院、当院の訪問診療が開始されました。今回は退院後3回目の診察です（あなたは1回目も2回目も対面で訪問診療しています）。 主介護者の長男嫁も同席しています。

【既往歴】上記の他、特記なし

【内服薬】アジルサルタン 20mg 1T 1×朝

【アレルギー歴】特記なし

【生活歴】飲酒：機会飲酒、喫煙：なし


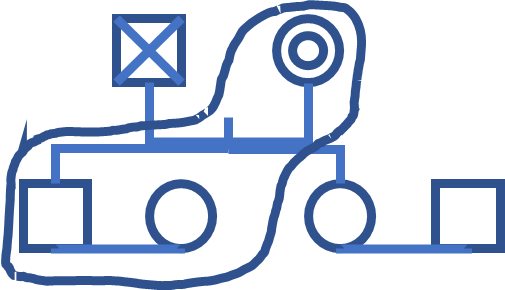


【家族状況】

・長男夫婦と同居、お嫁さんとの関係は良好

・ 孫はいない

・近くに長女夫婦も在住、介護には協力的

・長男夫婦、長女夫婦ともに農家

【バイタルサイン】（訪問看護師が実施）

血圧136/80 mmHg、脈拍60/分、体温 36.8℃、SpO2 98％ (room air)

高齢者総合的機能評価（comprehensive geriatric assessment：CGA）の視点でこの患者の診療を⾏ってください。時間は10分間です。

2024/2

シナリオ①：**患者役**シート

**課題**

あなたはH町にある無床診療所の医師です。診療所では外来診療の他に24時間体制の在宅診療を行っています。また、コロナ禍の収束後もオンライン診療に取り組んでいます。平日の午後（15時頃）、次の患者をD to P with N（訪問看護師が訪問した状態でのオンライン診療）で診療することになりました。患者宅は当院から片道40分かかるため、オンライン診療も織り交ぜながらの診療方針に本人・家族も同意しています。

**患者：82歳　女性　郡山 和美(こおりやま かずみ) さん**

高血圧で2か月に１回当院に通院していました。X-1年11月に外出中に転倒し、近隣のB総合病院に救急搬送、大腿骨頸部骨折の診断で入院しました。X年1月にリハビリを終えて自宅退院、当院の訪問診療が開始されました。今回は退院後3回目の診察です（あなたは1回目も2回目も対面で訪問診療しています）。 主介護者の長男嫁も同席しています。

【既往歴】上記の他、特記なし

【内服薬】アジルサルタン 20mg 1T 1×朝

【アレルギー歴】特記なし

【生活歴】飲酒：機会飲酒、喫煙：なし


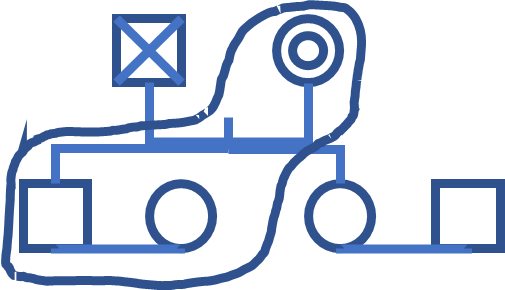


【家族状況】

・長男夫婦と同居、お嫁さんとの関係は良好

・ 孫はいない

・近くに長女夫婦も在住、介護には協力的

・長男夫婦、長女夫婦ともに農家

【バイタルサイン】（訪問看護師が実施）

血圧136/80 mmHg、脈拍60/分、体温 36.8℃、SpO2 98％ (room air)

高齢者総合的機能評価（comprehensive geriatric assessment：CGA）の視点でこの患者の診療を⾏ってください。時間は10分間です。

【看護師さん：キャラ設定】

オンライン診療のセッティングをしてくれている。（つつがなくオンラインは繋がり音声も良好）基本的には静かに患者とお嫁さんの話を聴いているため、画面にほとんど映らない。

【本人：キャラ設定】

やや抑うつ的な方をイメージしています。どんどん話すというよりは、少しずつ静かに思いを語る、そんな様子でお願いします。オンライン診療は初めてなのでやや動揺しており、「こうやって話したら先生にきこえるのかい？」「どこをみたらいいんだい？」などと最初はお嫁さんに聞いてください。

【お嫁さんキャラ設定】

どちらかといえば控えめな方で想定しています。ぐいぐい前に出てくる感じではありません。意見を求められたら語る感じにしてください。【家族状況】のところにお嫁さんの思いに関する記載があります。

【患者背景】

・リハビリをして退院はしたが転倒の不安が強くなりほとんど外出できていません。

・自宅内歩行は手すりにつかまりながらできていますが、ふらつきが強い状態です。自宅外は４点杖を用いるよう指導を受けましたが、ふらつきが強く自分だけでは外出できていません．退院後に外に出たのは週１回行き始めたデイサービスだけです。

・家業は農家で自宅隣の畑での農作業が楽しみでしたが、退院後はできていません。

【ご本人の思い】

解釈）今までのように歩いて外に出るのは難しいだろう。

期待）家族にはなるべく迷惑をかけたくない。

感情）畑仕事も友達とのお茶飲みもできず、生きがいを失ってしまった…。

畑に行けるだけでも何より嬉しいのだが…。

影響）畑仕事もできない。お茶のみ友達のところに出かけることもできなくなった。

【ADL】

BADL

Dressing 更衣：自立

Eating 食事：自立

Ambulation 移動：室内つかまり歩行、屋外は４点杖だがほぼ歩いていない。

Toileting 排泄：自立

Hygiene 衛生：自立

IADL

Shopping 買い物：家族がしている

Housekeeping清掃：家族がしている

Account 金銭管理：自立

Food preparation食事準備：家族がしている

Transport 公共交通：利用していない

AADL

・畑仕事が人生の楽しみであった。

・もう一つの楽しみが近所のお茶飲み友達の家に行き、友達と話すことだった。

・退院後はデイサービスに通い始めたが認知症の人が多く、馴染めていない。

【その他のCGA項目】

認知機能：Mini-Cog　3-item recall，Clock Drowing Testいずれも問題なし

運動機能：今回の骨折受傷前には転倒歴なし。1人で公共交通機関を利用し受診や買い物ができていた。骨折後は「廃用性」の筋力低下によるふらつきが強い。かなり気を付けているため退院後は転倒せず過ごせている。

視力・聴力：老眼鏡を使用、補聴器は使用せず問題なく聞き取りできている。

精神状態：今回の骨折を契機にやや抑うつ気分が認められている。

2質問法　ここ1か月、気分が落ち込んだり憂鬱な気分になっていることが多い：〇「そうですね、憂鬱な気持ちは多いです」

　　　　　ここ1か月、何をしても楽しくないと感じる：×？

「畑ができたら楽しいと思う」

GDS：6点、希死念慮：なし、妄想：なし

排尿・排泄：トイレに間に合わないことがあったため入院中からオムツを使用してい

る。過活動膀胱や排尿障害はなし。排便は問題ない。

睡眠：早く起きてしまうことは入院前からあるが、あまり変わらない。

食欲：食は以前から細いが食事量や食欲は変わっていない。偏食なし。

体重減少：なし

サポート：要介護１、デイサービスと訪問看護を週1回ずつ利用している。退院前に自宅内に手すりを設置し、自宅内ならつかまりながら自由に移動できる環境にある。同居の長男夫婦も近所の娘夫婦も農家なので、基本的に日中は不在だが自由に時間の調整はできる。通所、訪問リハビリの利用は現在はなし。

【長男嫁の思い】

・できれば以前のお義母さんのように元気になってほしい。

・お義母さんは畑仕事ができなくなって以来、元気がないことを心配している。

【評価のポイント】

・CGAをしていく中で「生きがいを失っている」ことに気づくことができる。

・抑うつに対して、２質問法やGDS15などの評価方法も適宜用いることができる
（下記参照）

・患者だけでなく家族の思いを引き出すことができる。

・適切に訪問や通所でのリハビリを提案し、「友人の家に行くこと」や「畑に行くこと」といった具体的な目標が、本人と医療者の間で立てることができる。

参考：老年期うつ病評価尺度（Geriatric depression scale 15；GDS15


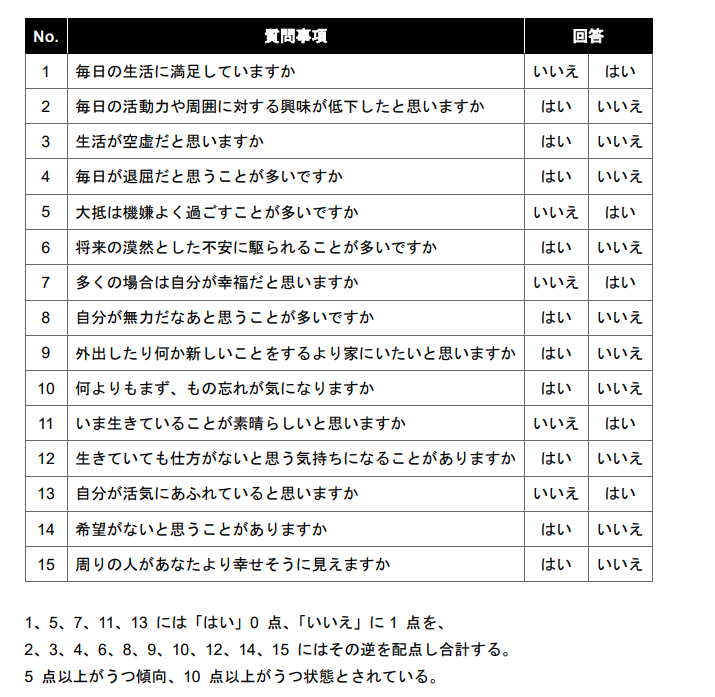


（日本老年医学会より　<https://www.jpn-geriat-soc.or.jp/tool/pdf/tool_11.pdf>）

2024/2

シナリオ②：**医師役**シート

**課題**

あなたはH町にある無床診療所の医師です。診療所では外来診療の他に24時間体制の在宅診療を行っています。また、コロナ禍の収束後もオンライン診療に取り組んでいます。平日の午後（15時頃）、次の患者をD to P with N（訪問看護師が訪問した状態でのオンライン診療）で診療することになりました。患者宅は当院から片道40分かかるため、オンライン診療も織り交ぜながらの診療方針に本人・家族も同意しています。

**患者：82歳　男性　小松 照三(こまつ てるぞう) さん**

高血圧で2か月に１回当院に通院していました。半年前に健診のレントゲンで異常陰影を指摘され、精査の結果、右肺小細胞癌の診断となりました。当初は抗がん剤を行いましたが、嘔気の副作用が強く、有害事象もあり、腰椎の骨転移も見つかったことから、これ以上の積極的な治療の適応にはならず、Best Supportive Careの方針となりました。先月退院し、当院の訪問診療が開始されました。今回は退院後3回目の診察です（あなたは1回目も2回目も対面で訪問診療しています）。 主介護者の妻も同席しています。

【既往歴】上記の他、特記なし

【内服薬】アジルサルタン 20mg 1T 1×朝、オキシコンチン(5mg) ２錠２× 8,20時、オキノーム散(2.5mg) 疼痛時頓用 1回１包、酸化マグネシウム(330mg) ６錠３× 毎食後

【アレルギー歴】特記なし

【生活歴】飲酒：機会飲酒、喫煙：半年前から禁煙（それまでは20本/日を20歳から）

【家族】夫婦2人暮らし。患者は長年庭師として働き、妻は主婦として夫を支えてきた。子供はおらず、遠方に患者の兄弟がいるが、お互い高齢で最近は疎遠になっている。

【その他】

・栄養：口から摂れなくなったら点滴はしてほしい。経腸栄養は希望しない。

・救急搬送：強い苦痛などが生じたときだけ希望

・最期を過ごす場所：保留

【バイタルサイン】（訪問看護師が実施）

血圧136/80 mmHg、脈拍60/分、体温 36.8℃、SpO2 98％ (room air)

前回の対面での訪問診療の最後に「今後の過ごし方、過ごす場所」の話になり、本人・妻ともに「次回までに考えておきます」とのことでした。この患者の診療を⾏ってください。

時間は10分間です。

2024/2

シナリオ②：**患者役**シート

**課題**

あなたはH町にある無床診療所の医師です。診療所では外来診療の他に24時間体制の在宅診療を行っています。また、コロナ禍の収束後もオンライン診療に取り組んでいます。平日の午後（15時頃）、次の患者をD to P with N（訪問看護師が訪問した状態でのオンライン診療）で診療することになりました。患者宅は当院から片道40分かかるため、オンライン診療も織り交ぜながらの診療方針に本人・家族も同意しています。

**患者：82歳　男性　小松 照三(こまつ てるぞう) さん**

高血圧で2か月に１回当院に通院していました。半年前に健診のレントゲンで異常陰影を指摘され、精査の結果、右肺小細胞癌の診断となりました。当初は抗がん剤を行いましたが、嘔気の副作用が強く、有害事象もあり、腰椎の骨転移も見つかったことから、これ以上の積極的な治療の適応にはならず、Best Supportive Careの方針となりました。先月退院し、当院の訪問診療が開始されました。今回は退院後3回目の診察です（あなたは1回目も2回目も対面で訪問診療しています）。 主介護者の妻も同席しています。

【既往歴】上記の他、特記なし

【内服薬】アジルサルタン 20mg 1T 1×朝、オキシコンチン(5mg) ２錠２× 8,20時、オキノーム散(2.5mg) 疼痛時頓用 1回１包、酸化マグネシウム(330mg) ６錠３× 毎食後

【アレルギー歴】特記なし

【生活歴】飲酒：機会飲酒、喫煙：半年前から禁煙（それまでは20本/日を20歳から）

【家族】夫婦2人暮らし。患者は長年庭師として働き、妻は主婦として夫を支えてきた。子供はおらず、遠方に患者の兄弟がいるが、お互い高齢で最近は疎遠になっている。

【その他】

・栄養：口から摂れなくなったら点滴はしてほしい。経腸栄養は希望しない。

・救急搬送：強い苦痛などが生じたときだけ希望

・最期を過ごす場所：保留

【バイタルサイン】（訪問看護師が実施）

血圧136/80 mmHg、脈拍60/分、体温 36.8℃、SpO2 98％ (room air)

前回の対面での訪問診療の最後に「今後の過ごし方、過ごす場所」の話になり、本人・妻ともに「次回までに考えておきます」とのことでした。この患者の診療を⾏ってください。

時間は10分間です。

【本人の設定】

右肺小細胞癌、腰椎転移（StageⅣ）の方です。まじめで気丈な性格です。以前は骨転移による体動時痛がありましたが、オキシコンチンの内服で自制内におさまっており、オキノーム散は最近は使用していません。他は特に症状はありません。入院生活と抗がん剤治療により、PS２（身の回りのことはできるが軽作業は難しい）までADLが低下してしまいました。本当は自宅で最期まで過ごしたいと考えていますが、高齢の妻に迷惑をかけたくないので、最期は病院への入院させてほしいと考えています。もし、医師から、今後の療養先についての希望を聞かれたら、「妻に迷惑をかけたくないので最期は病院へ入院させてください」と言ってください。背景や価値観、QOLについて十分に傾聴・聴取してもらえたら、在宅看取りについて前向きな姿勢を示してください。

＊オンライン診療は初めてですが、順応性がある方です。「こうやって話したら先生に聞こえるのかい？」、「どこを見たらいいんだい？」と妻に確認した後は特に問題なく診療に応じられます。

【看護師さん：キャラ設定】

オンライン診療のセッティングをしてくれている。（つつがなくオンラインは繋がり音声も良好）基本的には静かに患者とお嫁さんの話を聴いているため、画面にほとんど映らない。

【妻の設定】

昔ながらの、控え目で夫の一歩後ろを歩くような妻です。今後については夫の希望を優先したいと思っています。今後についての希望を聞かれて、患者が入院を希望したら、悩む様子を見せながらも「それが夫の希望なら…」と承諾してください。妻の状況について配慮され、介護サービスなどの支援について説明があれば、在宅看取りについて前向きな姿勢を示してください。妻はADL自立、認知機能良好、基礎疾患は変形性膝関節症のみです。

【患者の思い】

・めっきり体力が落ちて、最近は趣味の庭仕事をするのも身体がだるく、ほとんどできていない。せめて身の回りのことは頑張りたいが、最近はできない日もあり、落ち込むこともある。

・余命ははっきりと聞いていないが、そう遠くはないのだろう。

・本当は、妻と２人、自慢の庭が見える自宅で最期まで過ごしたいが、妻に迷惑をかけるくらいなら自分から病院に入院させてもらえるように頼もう。

・介護サービスについて詳しくは知らないが、妻の負担が軽くなるならお願いしたい。

・残される妻が心配だ。誰か相談に乗ってくれる人がいないものだろうか…。

【妻の思い】

・苦労しながらも、夫婦２人支え合って生きてきた。

・（夫が「最期は入院」と言ったことに対して）夫の希望を優先したい。ただ、それが真意でないなら（本当は家で最期を迎えたいなら）、本当は自宅で一緒に過ごしたい。

・自分も高齢で足腰が弱く、介護が必要になったときにひとりでやるには不安がある。

・経済的な心配はない。

【ADL】

BADL すべて自立。ADL低下に伴い、移動はゆっくり、入浴はシャワーが多くなった。

IADL 炊事・清掃・買い物は妻、内服管理は本人が行っている。

AADL 元庭師、自宅の庭も自分で剪定してきた。自慢の庭を夫婦で眺めるの楽しみ。

【その他のCGA項目】

認知機能：3-item recall　3/3，長谷川式認知機能検査 26/30点(逆数、計算で減点)

運動機能：疾患・廃用性によるサルコペニア　転倒歴はなし　杖使用なし

視力・聴力：眼鏡・補聴器は未使用　通常の会話で聞き取り可

精神状態：軽度抑うつ気分あり　興味関心の減退なし

排尿・排泄：オムツ使用なし　排尿障害・排便異常なし

サポート：要介護１、訪問看護を週1回利用している。

【評価のポイント】

最初は夫婦ともに病院への入院を希望しますが、これまでの背景やAADL、価値観を

聞いていくうちに、夫婦の望む希望は別にあることに気が付きます。

・どんな生活をしていきたいか、何を大事にして生きていくのかといった、価値観や希望を聞き出すことができる。

・患者だけでなく妻の思いを引き出すことができる。

・今後予想される妻の介護負担と対応する介護サービス等について適切に提案できる。

・初回のオンライン診療なので、医師の声が聞こえているか、画面上で医師が見えるか等の確認ができる。
